# Septins suppress the release of vaccinia virus from infected cells

**DOI:** 10.1083/jcb.201708091

**Published:** 2018-08-06

**Authors:** Julia Pfanzelter, Serge Mostowy, Michael Way

**Affiliations:** 1 Cellular Signalling and Cytoskeletal Function Laboratory, The Francis Crick Institute, London, England, UK; 2 Section of Microbiology, Medical Research Council Centre for Molecular Bacteriology and Infection, Imperial College London, London, England, UK; 3 Department of Immunology and Infection, London School of Hygiene & Tropical Medicine, London, England, UK

## Abstract

Septins are conserved components of the cytoskeleton that play important roles in many fundamental cellular processes including division, migration, and membrane trafficking. Septins can also inhibit bacterial infection by forming cage-like structures around pathogens such as *Shigella*. We found that septins are recruited to vaccinia virus immediately after its fusion with the plasma membrane during viral egress. RNA interference–mediated depletion of septins increases virus release and cell-to-cell spread, as well as actin tail formation. Live cell imaging reveals that septins are displaced from the virus when it induces actin polymerization. Septin loss, however, depends on the recruitment of the SH2/SH3 adaptor Nck, but not the activity of the Arp2/3 complex. Moreover, it is the recruitment of dynamin by the third Nck SH3 domain that displaces septins from the virus in a formin-dependent fashion. Our study demonstrates that septins suppress vaccinia release by “entrapping” the virus at the plasma membrane. This antiviral effect is overcome by dynamin together with formin-mediated actin polymerization.

## Abstract (precis)

Septins play important roles in many cellular processes. Pfanzelter et al. show that septins suppress the release of vaccinia virus from infected cells by trapping virions on the plasma membrane. This antiviral effect is overcome by dynamin and formin-mediated actin polymerization.

## Graphical Abstract

**Figure:**
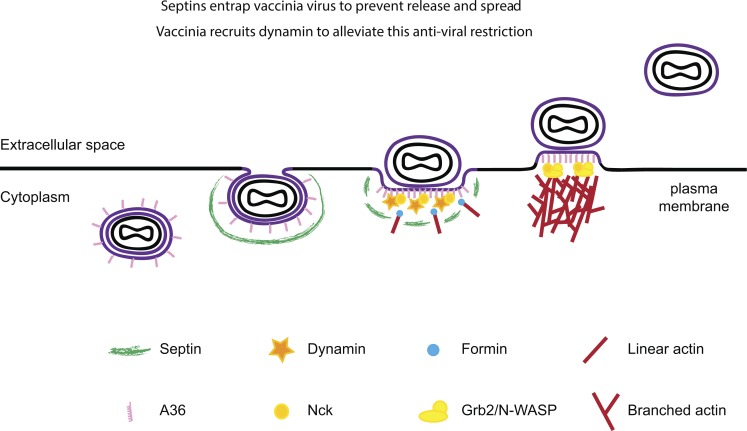

## Introduction

**Figure 1.**
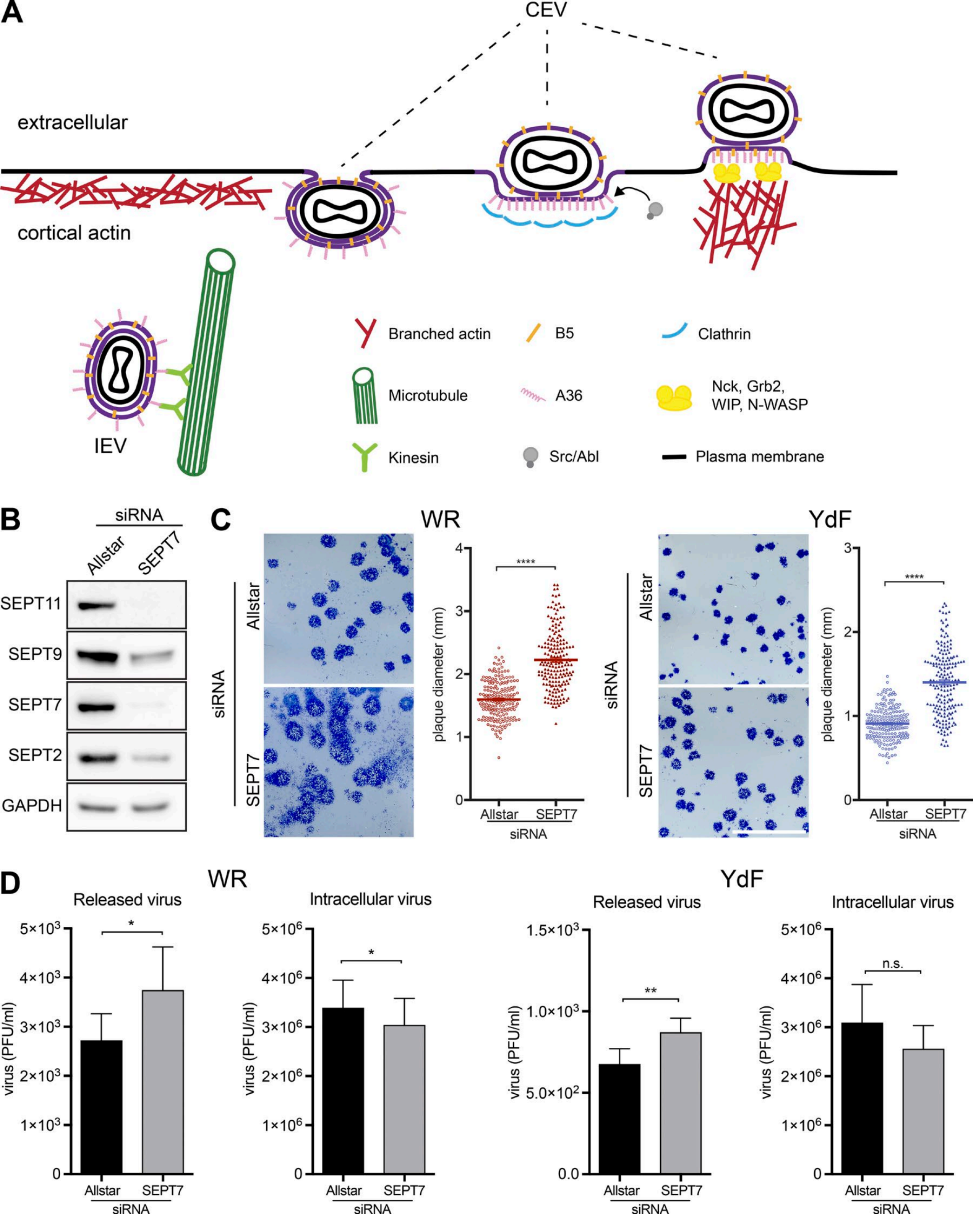
**Loss of septin promotes virus release and spread. (A)** Schematic depicting key events at the plasma membrane during vaccinia virus egress. A36, the actin tail nucleator phosphorylated by Src and Abl family kinases, and B5, exposed on the surface of CEV, are integral viral membrane proteins. **(B)** Immunoblot analysis confirms that SEPT7 siRNA treatment of A549 cells for 72 h leads to loss of SEPT7 as well as reduction in the levels of SEPT2, SEPT9, and SEPT11. **(C)** Images of plaques formed on A549 cell monolayers treated with control (Allstar) or SEPT7 siRNA for 72 h under liquid overlay after 3 d of infection with WR or the YdF strain of virus, which is deficient in actin tail formation. Plaque comets are seen as a diffuse spray emanating from WR plaques in liquid overlay conditions. The graphs show the quantification of plaque size (*n* > 190) with error bars representing the SEM from three independent experiments. **(D)** Quantification of virus release and total intracellular virus in A549 cells in the presence or absence of SEPT7 at 18 h after infection with WR or the YdF virus. Error bars represent SEM from three independent experiments. *, P < 0.05; **, P < 0.01; ****, P < 0.0001. Bar, 1 cm.

During vaccinia virus infection, the newly assembled intracellular enveloped virus (IEV) is transported on microtubules to the plasma membrane by kinesin-1 (Hollinshead et al., 2001; Rietdorf et al., 2001; Ward and Moss, 2001; Dodding et al., 2011; Leite and Way, 2015; Fig. 1 A). At the cell periphery, IEVs fuse with the plasma membrane by an unknown mechanism after crossing the actin cortex (Arakawa et al., 2007a) to release infectious virus into the surrounding environment (Leite and Way, 2015). Not all virions, however, are immediately released, as some remain attached to the outside of the cell and are referred to as cell-associated enveloped viruses (CEVs; Fig. 1 A; Leite and Way, 2015). These virions are able to signal back into the cell to induce Arp2/3-dependent actin polymerization to enhance their spread into adjacent cells (Cudmore et al., 1995, 1996; Frischknecht et al., 1999; Hollinshead et al., 2001; Ward and Moss, 2001; Doceul et al., 2010). CEVs achieve this feat by stimulating Src and Abl kinase–mediated phosphorylation of tyrosine 112 and 132 of A36, an integral viral membrane protein that becomes localized beneath a CEV after the virus fuses with the plasma membrane (Fig. 1 A; Frischknecht et al., 1999; van Eijl et al., 2000; Scaplehorn et al., 2002; Newsome et al., 2004, 2006; Reeves et al., 2005). Phosphorylation of tyrosine 112 leads to the association of Nck, resulting in the recruitment of a complex of WIP and N-WASP that locally activates Arp2/3-mediated actin polymerization (Frischknecht et al., 1999; Moreau et al., 2000; Zettl and Way, 2002; Weisswange et al., 2009; Donnelly et al., 2013). Phosphorylation of tyrosine 132 of A36 is not essential for CEV-induced actin polymerization, but its presence makes the process more efficient by recruiting Grb2 to help stabilize the Nck, WIP, and N-WASP signaling network (Scaplehorn et al., 2002; Weisswange et al., 2009; Donnelly et al., 2013). In addition, the RhoGTPase Cdc42, an activator of N-WASP, also helps facilitate CEV-induced actin polymerization (Humphries et al., 2014). Cdc42 is locally activated by the RhoGEF intersectin-1, which is recruited to CEV by interacting with three Asn-Pro-Phe (NPF) motifs toward the C terminus of A36 (Humphries et al., 2014; Snetkov et al., 2016). Intersectin-1 also recruits clathrin to CEV via its adaptor AP-2 before actin tail formation (Fig. 1 A; Humphries et al., 2012; Snetkov et al., 2016). AP-2 and clathrin do not remain associated with CEV when actin polymerization is stimulated. Nevertheless, their transient recruitment helps polarize A36 and N-WASP beneath CEV, leading to more rapid and sustained actin polymerization, which enhances the cell-to-cell spread of vaccinia (Humphries et al., 2012).

Although we have a good molecular understanding of how CEVs induce actin polymerization, we still lack crucial insights into the events taking place when IEVs fuse with the plasma membrane during viral spread. Previous observations from genome-wide high-throughput RNAi-based screens demonstrate that knockdown of septins enhances vaccinia replication and/or spread (Sivan et al., 2013; Beard et al., 2014). Septins are a family of cytoskeletal proteins found in animals and fungi (Mostowy and Cossart, 2012). In humans, there are 13 septins, which are subdivided into four different homology groups (SEPT2, SEPT3, SEPT6, and SEPT7; Saarikangas and Barral, 2011; Mostowy and Cossart, 2012; Neubauer and Zieger, 2017). Septins form heterooligomers that assemble into nonpolar filaments and ring-like structures in the cytoplasm and on the plasma membrane (Kinoshita et al., 2002; Sirajuddin et al., 2007; Bertin et al., 2008; Bridges et al., 2014). All higher-order septin structures contain SEPT2 and SEPT6 family members but are critically dependent on SEPT7 (Sirajuddin et al., 2007). Septins play a variety of roles in many cellular processes including cell division and migration as well as membrane trafficking by virtue of their ability to associate with lipids, microtubules, and actin filaments (Saarikangas and Barral, 2011; Mostowy and Cossart, 2012). Septins can also inhibit bacterial infection by forming cage-like structures around intracellular pathogens such as *Shigella flexneri* (Mostowy et al., 2010; Sirianni et al., 2016). We now report that septins are recruited to vaccinia virus after its fusion with the plasma membrane and act to suppress the release of the virus from infected cells. Moreover, the Nck-mediated recruitment of dynamin to the virus as well as formin-driven actin polymerization displaces septins, thereby overcoming their antiviral effect.

## Results

### Septins suppress the release and cell-to-cell spread of vaccinia

To understand the role of septins during vaccinia infection, we examined the impact of the loss of SEPT7 on the release and spread of the Western Reserve (WR) strain of vaccinia virus. The knockdown efficiency of SEPT7, which is essential for septin filament formation and function (Sirajuddin et al., 2007), was confirmed by immunoblot analysis (Fig. 1 B). We found that loss of SEPT7 leads to a significantly larger WR plaque diameter in confluent A549 cell monolayers with liquid (Fig. 1 C) or semisolid overlay (Fig. S1, A and B); the latter measures only direct cell-to-cell spread. It is also striking that loss of SEPT7 leads to the formation of extensive plaque comets in liquid overlay, which are seen as a diffuse spray emanating from a central round plaque. This phenomenon is indicative of enhanced virus release in liquid overlay conditions (Yakimovich et al., 2015). In agreement with their assembly into functional heteromeric complexes, we found that RNAi-mediated loss of SEPT2 or SEPT11 also increases the size of plaques induced by WR (Fig. S1 C). The increase in plaque size in the absence of SEPT7 is not restricted to WR, as it is also apparent in cells infected with WR expressing A36-YdF (designated as YdF), a vaccinia strain deficient in actin tail formation and cell-to-cell spread since A36 cannot be phosphorylated on tyrosine 112 or 132 (Rietdorf et al., 2001; Ward and Moss, 2001; Fig. 1 C and Fig. S1 A). In both cases, there was also a concomitant increase in virus release when SEPT7 was depleted (Fig. 1 D). This increase in release is not related to virus production, as septin loss actually reduces the number of intracellular virions (Fig. 1 D).

**Figure 2.**
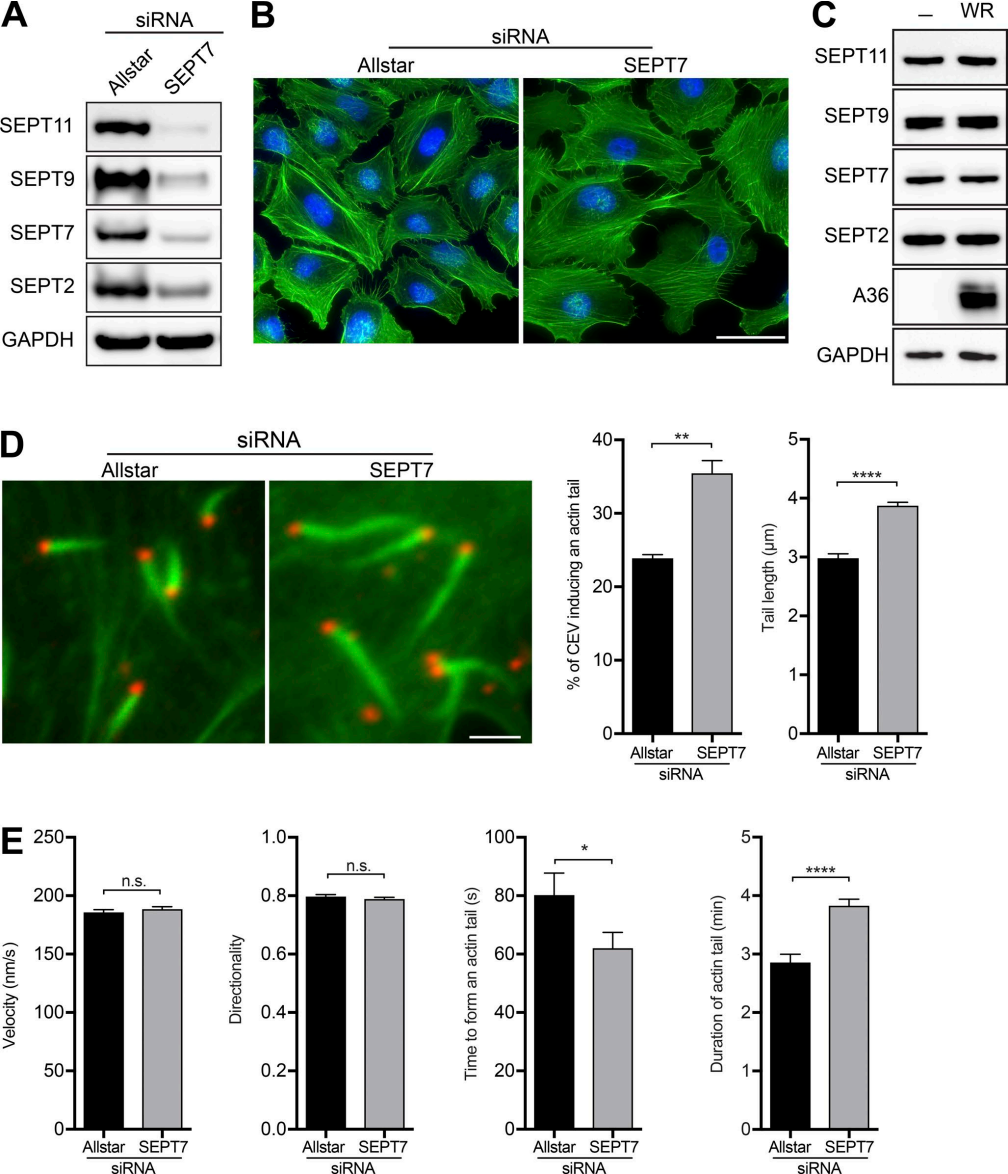
**Septin suppresses actin tail formation. (A)** Immunoblot analysis confirms that SEPT7 siRNA treatment of HeLa cells for 72 h reduces the levels of SEPT7, SEPT2, SEPT9, and SEPT11. **(B)** Representative immunofluorescence images showing that loss of SEPT7 in uninfected HeLa cells has no obvious impact on the actin cytoskeleton (green). Bar, 50 µm. **(C)** Immunoblot analysis confirming that infection of HeLa cells with WR vaccinia virus does not alter the level of SEPT2, SEPT7, SEPT9, or SEPT11. **(D)** Representative images of actin tails (green) induced by WR (red labeled with B5 antibody) in HeLa cells treated with Allstar (control) or SEPT7 siRNA. Bar, 2 µm. The graphs show the quantification of the number of CEVs inducing actin tails and their length. Error bars represent SEM from three independent experiments in which a total of 30 cells were analyzed for actin tail number and 370 tails were measured for length. **(E)** Quantification of the velocity, directionality, time to form, and duration of actin tails in the absence of SEPT7. Error bars represent SEM from three independent experiments in which a total of >150 actin tails were measured for duration, >50 events for tail initiation from four independent experiments, and >300 actin tails for speed and directionality. *, P < 0.05; **, P < 0.01; ****, P < 0.0001.

RNAi-mediated depletion of SEPT7 in HeLa cells has no appreciable impact on the actin cytoskeleton (Fig. 2, A and B), and vaccinia infection does not affect the level of septin expression (Fig. 2 C). However, loss of SEPT7 increases the number of CEV inducing actin tails (35.5 ± 1.7% compared with 23.9 ± 0.5%), which are also significantly longer (3.9 ± 0.1 µm compared with 3.0 ± 0.1 µm; Fig. 2 D and Fig. S2 A). Loss of SEPT2, SEPT9, or SEPT11 also results in more CEV-inducing actin tails that are again longer than normal (Fig. S2 B). The velocity and directionality of actin tails remained the same in the absence of SEPT7 and functional septins (Fig. 2 E). However, the time required for actin tail formation after the virus reached the cell periphery decreased (62.1 ± 5.4 s compared with 80.2 ± 7.5 s). It was also noticeable that actin tails had a significantly longer lifetime, lasting on average 3.8 ± 0.1 min compared with 2.9 ± 0.1 min (Fig. 2 E). Our results demonstrate that septins, rather than inhibiting earlier steps in virus replication, exert their negative effect on vaccinia by suppressing actin tail formation, viral release, and cell-to-cell spread.

### Septins are recruited by CEV before actin polymerization

**Figure 3.**
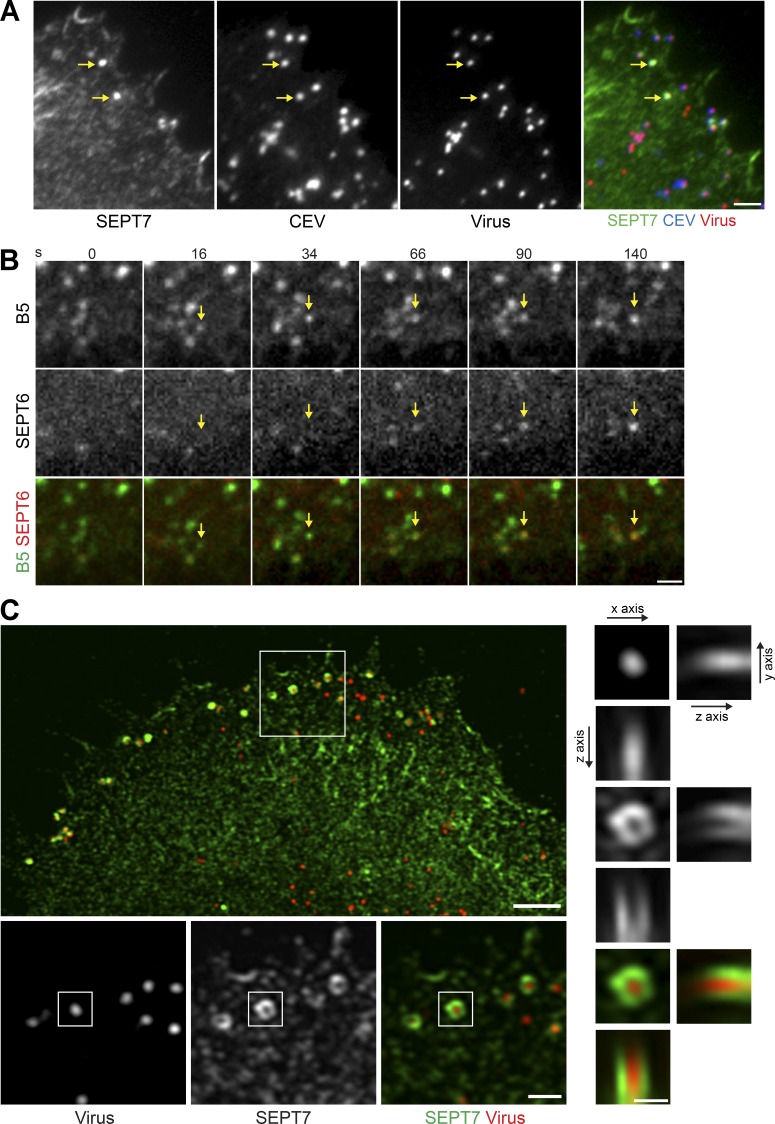
**Septins are only recruited by CEV on the cell surface. (A)** Representative immunofluorescence image showing the association of SEPT7 with CEV (yellow arrows) detected with the B5 antibody before membrane permeabilization in HeLa cells infected with WR expressing A3-RFP (virus) for 8 h. **(B)** Video stills showing GFP-SEPT6 (SEPT6) recruitment to an extracellular virus detected with the Alexa Fluor 488–conjugated B5 antibody (B5; yellow arrows) in live cells. The time in seconds is indicated. See Video 1. **(C)** Representative Airyscan confocal immunofluorescence image showing the association of SEPT7 (green) around CEV (red) in HeLa cells infected with WR expressing A3-RFP for 8 h. Bars: (A and B) 2 µm; (C) 3 µm (large upper image), 1 µm (lower medium-sized boxed images), and 300 nm (right small boxed images).

Immunofluorescence analysis of HeLa cells at 8 h after infection reveals that endogenous SEPT7 colocalizes with extracellular CEV in the cell periphery (Fig. 3 A). Endogenous SEPT2, SEPT9, and SEPT11 are also recruited to virus in the cell periphery (Fig. S3 A). Live imaging of infected cells in media containing an Alexa Fluor 488–conjugated B5 antibody that detects CEV after IEV fuse with the plasma membrane (Fig. 1 A; Humphries et al., 2012) reveals that mCherry-SEPT6 only accumulates on CEVs that are labeled with the Alexa Fluor 488–conjugated B5 antibody (Fig. 3 B and Video 1). Septins are therefore recruited to viral particles only after they fuse with the plasma membrane. Quantification reveals that septins are recruited to the virus 31.0 ± 2.4 s (*n* = 45) after the virus becomes stationary following microtubule-based transport to the cell periphery. Consistent with their role in suppressing virus release from the plasma membrane, Airyscan confocal imaging reveals that septins wrap around CEV, forming cage-like structures (Fig. 3 C). To examine the stability of the septin “cage,” we performed FRAP analysis. We found that the half-life of recovery of GFP-SEPT6 on CEV in WR-infected cells was 27.42 ± 1.4 s (Fig. S3 B). However, in contrast to the signaling network responsible for inducing actin polymerization (Weisswange et al., 2009; Humphries et al., 2014), the recovery is only 54.18 ± 1.48% and not complete.

**Figure 4.**
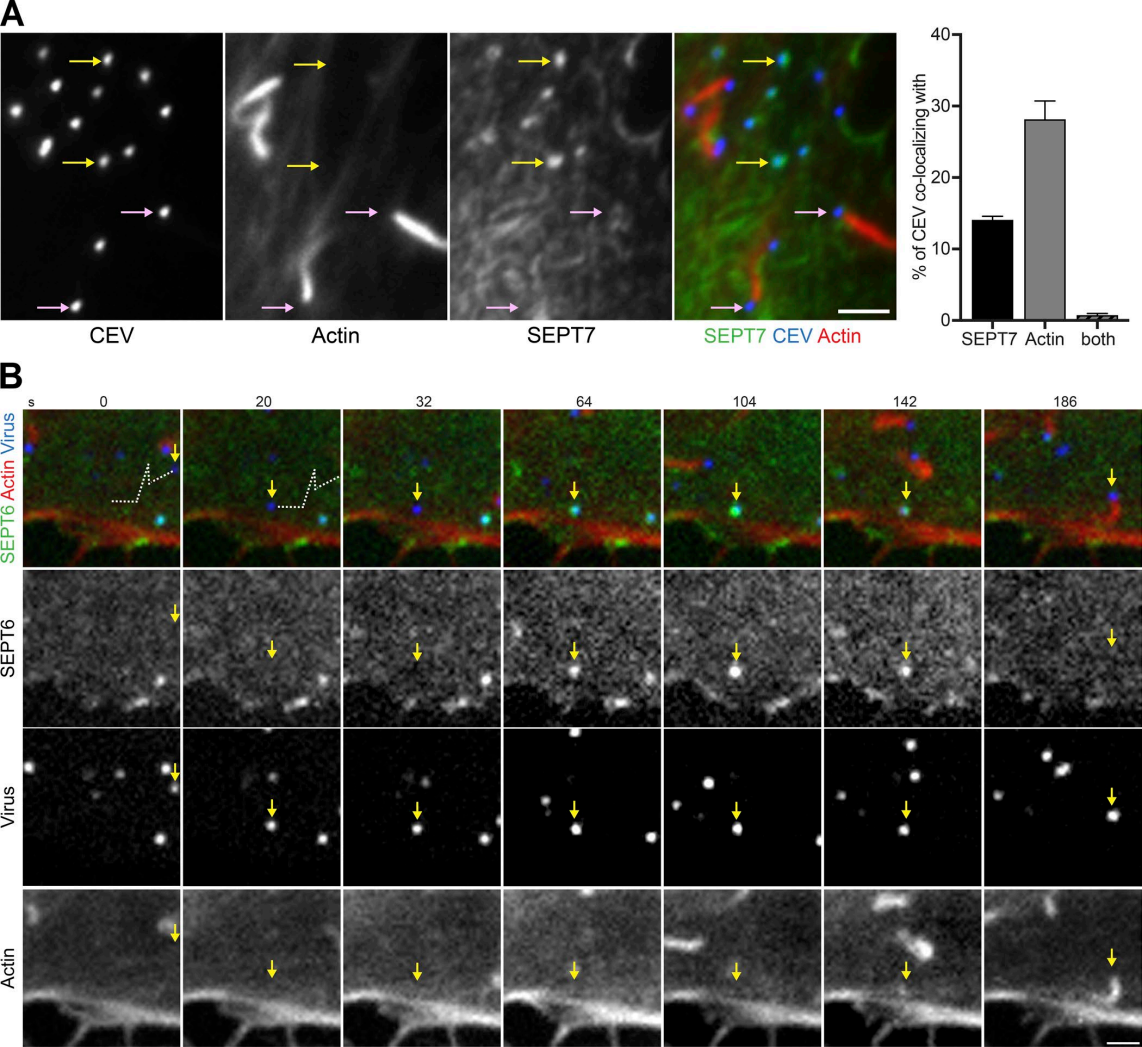
**Septin is recruited transiently to CEV before actin tail formation. (A)** Immunofluorescence analysis reveals CEV, detected with the B5 antibody before membrane permeabilization, either recruit septin (yellow arrows) or induce actin tails (pink arrows) but not both. The graph shows the quantification the percentage of CEV colocalizing with SEPT7 or inducing actin tails. Error bars represent SEM from three independent experiments in which 900 CEVs were analyzed in 30 cells. **(B)** Video stills from live cell imaging of HeLa cells expressing LifeAct-iRFP (Actin) and GFP-SEPT6 and infected with WR encoding A3-RFP (virus). SEPT6 is recruited to CEV but is lost when actin polymerization starts. The white dotted line corresponds to the initial microtubule-dependent movement of virion to the cell periphery. The time in seconds is indicated. See Video 2. Bars, 2 µm.

Interestingly, SEPT7 is only associated with 13.3 ± 1.9% of CEV (Fig. 4 A). Furthermore, the presence of septins is mutually exclusive to actin tails, which are associated with 23.4 ± 2.6% of CEV (Fig. 4 A). This suggests that septin recruitment and actin polymerization are temporally distinct processes or that there are two CEV populations with one recruiting septins and the other inducing actin tails. To establish which scenario is correct, we performed live cell imaging on HeLa cells stably expressing GFP-SEPT6 and Lifeact-iRFP that are infected with WR encoding A3-RFP to visualize virions. We found that septins are recruited to vaccinia before actin tail formation (Fig. 4 B and Video 2). Moreover, septins are lost from the virus when actin polymerization is initiated. Interestingly, septins can return to CEV if actin-based motility ceases (Fig. S3 C and Video 3).

### Septin and clathrin are recruited independently of each other

**Figure 5.**
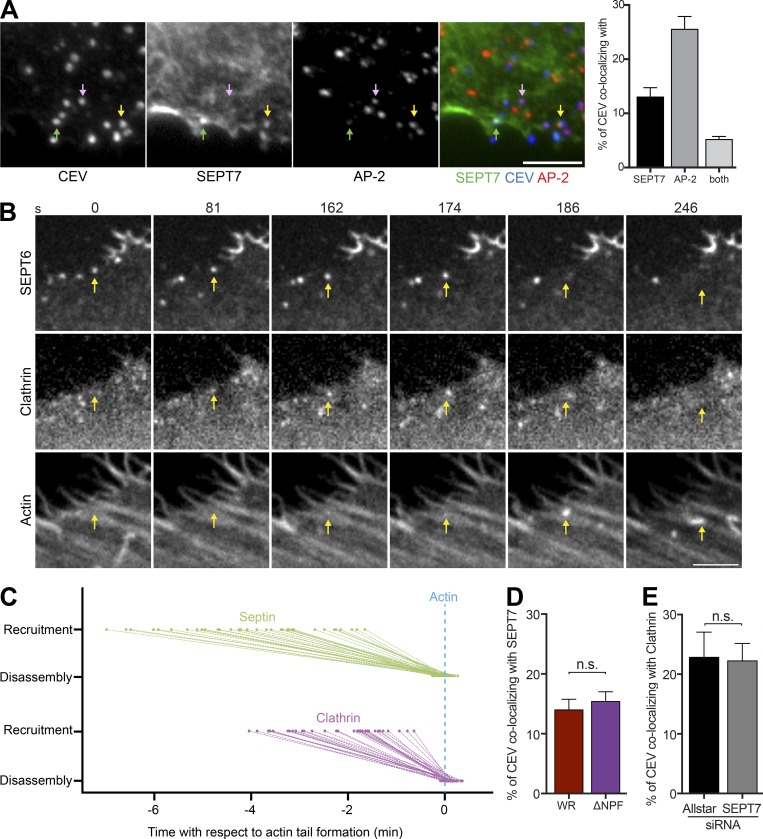
**Septin and clathrin are recruited independently to CEV. (A)** Immunofluorescence images showing the association of SEPT7 (green arrow), AP-2 (pink arrow), or both proteins (yellow arrow) with CEV (detected with the B5 antibody before membrane permeabilization) in HeLa cells infected with WR for 8 h. The graph shows percentage of CEV with SEPT7, AP-2, or both proteins. **(B)** Video stills taken from live cell imaging of WR-infected HeLa cells expressing GFP-SEPT6, mCherry-clathrin light chain (Clathrin), and LifeAct-iRFP (Actin). SEPT6 is recruited to CEV before clathrin, but both proteins are lost when actin polymerization starts. The time in seconds is indicated. See Video 4. **(C)** The graph shows the recruitment and loss of SEPT6 and clathrin to CEV with respect to actin tail formation. **(D)** The graph shows the percentage of CEV recruiting septin in the presence (WR) or absence (ΔNPF) of clathrin. **(E)** The percentage of CEV recruiting clathrin in the presence (Allstar) or absence of SEPT7. Bars, 5 µm. Error bars represent SEM from three independent experiments. For protein timings in C, 36 virus particles were analyzed, and for each of the other conditions, 900 CEVs were analyzed from 30 cells.

We have previously demonstrated that AP-2 and clathrin are recruited to CEV before actin tail nucleation (Humphries et al., 2012). Clathrin, like septins, is also lost from the virus when CEVs induce actin polymerization. Given the similarities, we investigated whether there is any functional relationship between clathrin and septins before actin tail formation. Immunofluorescence analysis reveals that at 8 h after infection, 13.1 ± 1.6% and 25.6 ± 2.3% of CEVs recruit SEPT7 and AP-2, respectively, whereas 5.2 ± 0.5% of viruses have both proteins (Fig. 5 A). This overlap suggests that septins and clathrin are recruited sequentially. To determine the temporal order of their recruitment, we performed live cell imaging of WR-infected HeLa cells stably expressing GFP-SEPT6 and Lifeact-iRFP that had also been transiently transfected with mCherry-tagged clathrin light chain (clathrin; Fig. 5 B and Video 4). We found that SEPT6 and clathrin are recruited 4.0 ± 0.2 min and 2.2 ± 0.2 min, respectively, before actin nucleation (Fig. 5 C). As actin polymerization is initiated, septins are rapidly lost from the virus (0.79 ± 1.1 s) before clathrin (5.70 ± 0.98 s; Fig. 5 C). This suggests that septins may help promote recruitment of clathrin. However, in rare cases, the virus recruits septin but not clathrin before actin tail formation, raising the possibility that septins and clathrin can act independently of one another (Fig. S4 A and Video 5).

To test this hypothesis, we took advantage of WR expressing A36 ΔNPF1-3, which is deficient in AP-2 and clathrin recruitment as A36 lacks the three NPF motifs required for their recruitment via intersectin (Snetkov et al., 2016). We found that the inability to recruit clathrin does not impact on the number of CEVs with septin (13.8 ± 1.6% and 15.46 ± 1.5% for WR and ΔNPF1-3, respectively; Fig. 5 D). Conversely, RNAi-mediated depletion of SEPT7 did not change the number of CEVs recruiting clathrin (22.9 ± 4.1% and 22.3 ± 2.9% for control and SEPT7 knockdown, respectively; Fig. 5 E). These data suggest that septins and clathrin are recruited independently and do not appear to influence each other during vaccinia egress.

### Arp2/3-driven actin polymerization does not promote loss of septin

**Figure 6.**
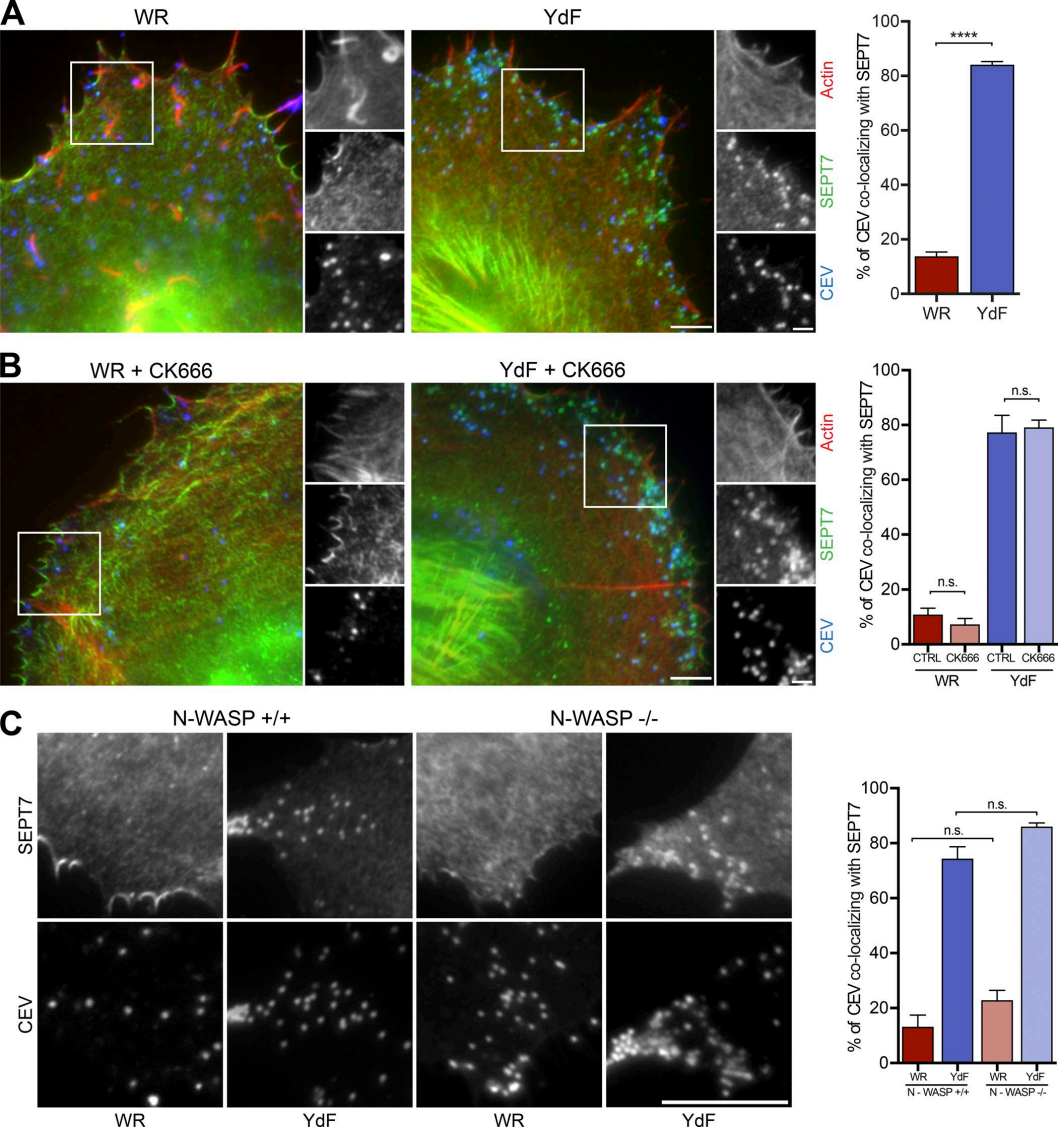
**Arp2/3-driven actin polymerization does not promote septin loss. (A)** Immunofluorescence images showing the association of SEPT7 (green) with CEV, detected with the B5 antibody before membrane permeabilization (blue) in HeLa cells infected with WR and the YdF virus, which is deficient in actin tail formation (red) at 8 h. The graph shows the percentage of CEV recruiting SEPT7 for the two different viruses. (**B)** Images showing the association of SEPT7 with CEV in HeLa cells infected with WR or YdF and treated with the Arp2/3 inhibitor CK666. The graph shows the percentage of CEV with SEPT7 in the presence (CK666) and absence (CTRL) of the Arp2/3 inhibitor for the two different viruses. **(C)** Images showing the association of SEPT7 with CEV in N-WASP parental and knockout MEFs infected with WR and YdF for 10 h. The graph shows the percentage of CEV colocalizing with SEPT7 for the indicated viruses in the presence (+/+) or absence (−/−) of N-WASP. Error bars represent SEM from three independent experiments in which 900 virus particles were analyzed across 30 cells. Bars: (A and B) 5 µm; (A and B, insets) 2 µm. ****, P < 0.0001.

Our previous observations demonstrated that a lack of actin polymerization increases the number of CEVs with AP-2 and clathrin (Humphries et al., 2012). We therefore investigated if the same holds true for septins, given they are also lost as actin accumulates on the virus. Strikingly, we found that the association of SEPT7 with CEV increases to 84.1 ± 1.1% in cells infected with the A36-YdF virus, which cannot induce actin tails (Fig. 6 A). FRAP analysis, however, reveals that the dynamics of GFP-SEPT6 on the A36-YdF virus in the absence of actin polymerization is similar to that seen with WR (Fig. S3 B).

The dramatic increase in the number of A36-YdF viruses with septin compared with WR suggests that actin tail formation inhibits septin recruitment and/or promotes its displacement from the virus. To further explore this possibility, we treated cells with CK666 to inhibit the ability of the virus to induce Arp2/3-dependent actin polymerization. Unexpectedly, treatment of infected cells with CK666 had no significant impact on the number of CEVs recruiting SEPT7 during WR or A36-YdF virus infection (Fig. 6 B). To confirm that the presence of SEPT7 on CEV is not influenced by Arp2/3-mediated actin polymerization, we infected N-WASP^−/−^ mouse embryonic fibroblasts (MEFs), which are deficient in actin tail formation (Snapper et al., 2001; Weisswange et al., 2009). As with chemical inhibition of Arp2/3, we observed that the presence or absence of N-WASP did not impact the ability of CEV to recruit SEPT7 (Fig. 6 C). This suggests that Arp2/3-mediated actin tail formation per se does not displace septins from the virus.

### Nck negatively regulates septin recruitment

**Figure 7.**
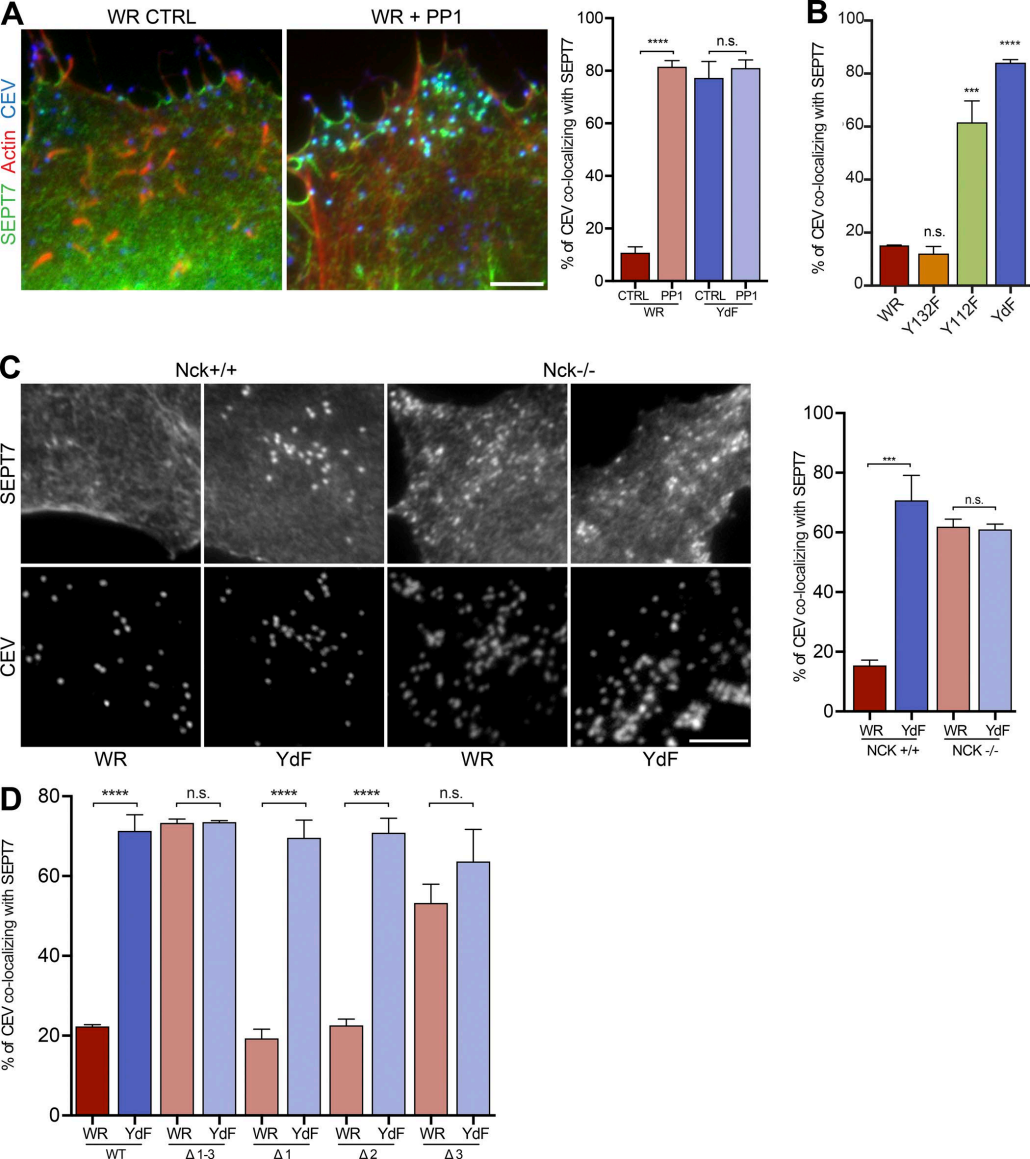
**A36 phosphorylation and recruitment of Nck displaces septins. (A)** Representative images showing the association of SEPT7 with CEV in WR-infected HeLa cells in the presence or absence (CTRL) of PP1. The graph shows the percentage of CEV in WR- and YdF-infected cells recruiting SEPT7. **(B)** The percentage of CEV recruiting SEPT7 in HeLa cells infected with vaccinia expressing A36 (WR), A36-Y132F (Y132F), A36-Y112F (Y112F), and A36-YdF (YdF). **(C)** Immunofluorescence images showing the recruitment of SEPT7 to CEV in Nck parental and knockout MEFs infected with WR and YdF virus for 20 h. The graph shows the percentage of CEV with SEPT7 for the indicated viruses in the presence (+/+) or absence (−/−) of Nck. **(D)** Quantification of the percentage of CEV recruiting SEPT7 in Nck knockout MEFs stably expressing GFP-Nck or the indicated function disrupting SH3 domain point mutants. Error bars represent SEM from three independent experiments in which a total of 900 CEVs were analyzed in 30 cells. Bars, 5 µm. ***, P < 0.001; ****, P < 0.0001.

How can the strong increase in SEPT7 recruitment to the A36-YdF virus be explained? The A36-YdF virus does not induce actin tails because A36 cannot be phosphorylated on tyrosine 112 and 132. Nevertheless, the A36-YdF virus still activates Src and Abl family tyrosine kinases (Newsome et al., 2004). This raises the possibility that it is the phosphorylation of A36 that promotes septin loss from the virus. To explore this hypothesis, we treated WR-infected cells with the Src and Abl family kinase inhibitor PP1. We found that treatment of cells with PP1 increases the number of CEV with SEPT7 in WR infection to the same extent as seen with the A36-YdF virus (Fig. 7 A). The fact that PP1 treatment had no effect on septin recruitment to the A36-YdF virus suggests that their presence is regulated by phosphorylation of tyrosine 112 and/or 132 of A36 or by the subsequent recruitment of Nck and/or Grb2. To begin to distinguish between these possibilities, we examined the level of SEPT7 recruitment to CEV in cells infected with WR expressing A36-Y112F or A36-Y132F, which are deficient in Nck and Grb2 recruitment, respectively (Scaplehorn et al., 2002). We found that the A36-Y132F virus had low levels of SEPT7 recruitment comparable to WR, indicating that Grb2 does not influence septins on the virus (Fig. 7 B). In contrast, the A36-Y112F virus, like A36-YdF, had high levels of SEPT7 recruitment. To determine whether SEPT7 recruitment is regulated by phosphorylation of tyrosine 112 of A36 or the presence of Nck, we infected Nck^−/−^ MEFs lacking both Nck1 and Nck2 (Bladt et al., 2003) with WR or the A36-YdF virus. We found that SEPT7 recruitment to CEV was equally high for both viruses in the absence of Nck (Fig. 7 C). In contrast, SEPT7 is only present on large numbers of CEV in the parental Nck^+/+^ cells infected with the A36-YdF virus. This demonstrates that it is the recruitment of Nck by phosphorylated tyrosine 112 of A36 that negatively influences the recruitment of septins to CEV. This explains why septin recruitment remains low in the absence of N-WASP (Fig. 6 C), as CEVs still recruit Nck in WR-infected N-WASP^−/−^ MEFs (Weisswange et al., 2009).

It is possible that the recruitment of Nck displaces septins because there are steric clashes beneath the CEV. Alternatively, Nck might recruit an additional factor via its SH3 domains that then promote septin dissociation. To distinguish between these two possibilities, we infected Nck^−/−^ cells stably expressing GFP-tagged Nck1 mutants with defective SH3 domains (Donnelly et al., 2013). We found that functional disrupting point mutations in all three Nck1 SH3 domains increases the number of WR virions with SEPT7 to the level seen for the A36-YdF virus (Fig. 7 D). This suggests that it is not spatial constraints but rather SH3 domain-dependent recruitment of a protein that promotes the displacement of septins from the virus. Interestingly, mutation of the third but not the first or second Nck SH3 domain increases SEPT7 recruitment to WR (Fig. 7 D). This suggests that the third SH3 domain of Nck is recruiting at least one protein that is responsible for displacing septins from the virus.

### Dynamin promotes septin dissociation from CEV

**Figure 8.**
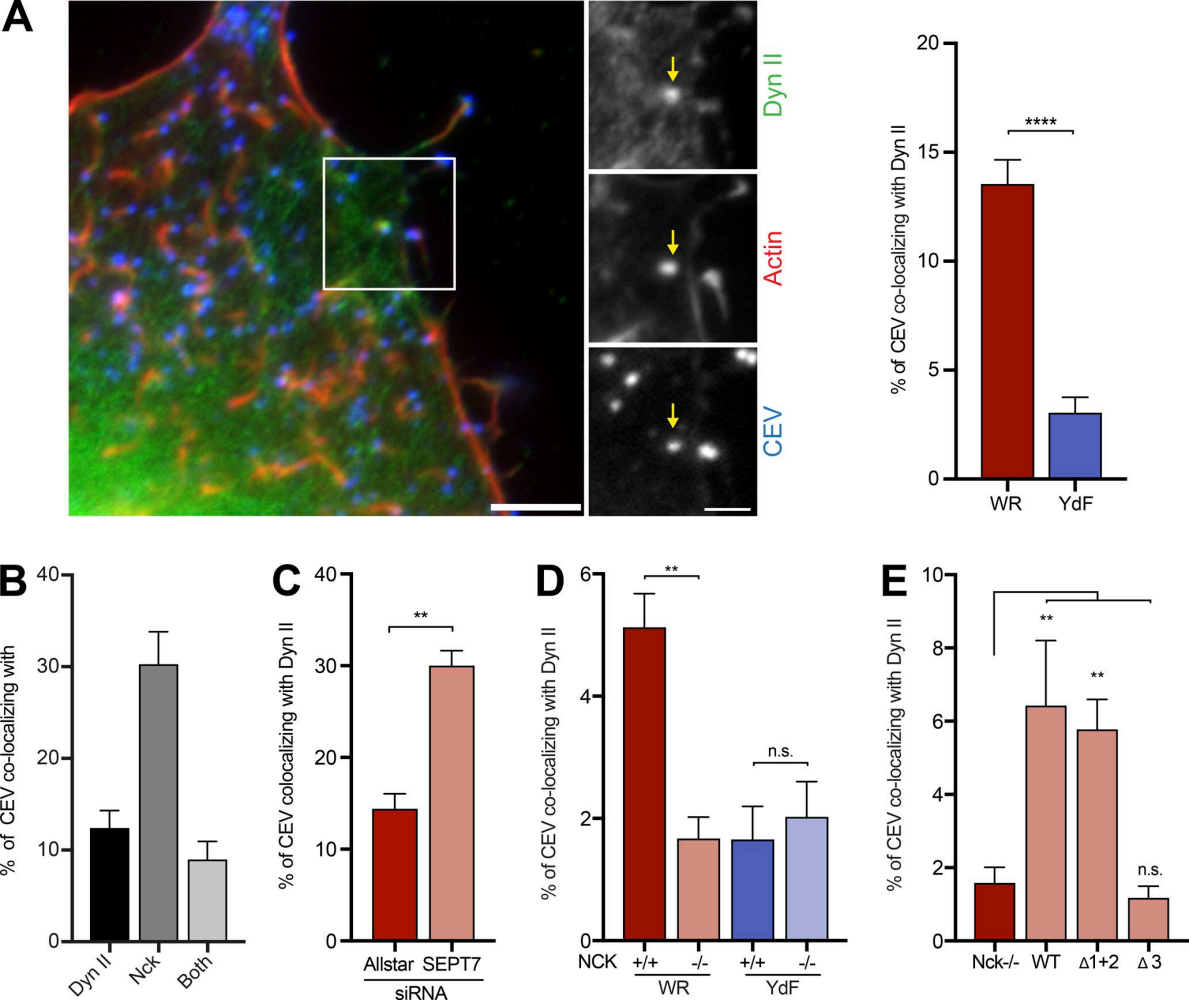
**Nck recruits dynamin to CEV. (A)** Dyn II and actin are recruited to CEV in HeLa cells infected with WR. The graph shows the percentage of CEV recruiting dyn II in WR- and YdF-infected cells. **(B)** The graph shows the percentage of CEV with dynamin, Nck, or both proteins in HeLa cells infected with WR. **(C)** Quantification of the percentage of CEV recruiting dyn II in the presence (Allstar) or absence (SEPT7) of septin in WR-infected HeLa cells. **(D)** The graph shows the percentage of CEV recruiting dynamin in Nck parental (+/+) and knockout cells (−/−) infected with WR or the YdF virus. **(E)** The graph shows the percentage of CEV recruiting dyn II in WR-infected Nck knockout cells (−/−) expressing GFP-tagged Nck (WT) or mutants with defective first and second SH3 (Δ1+2) or third SH3 (Δ3) domains. Error bars represent SEM from three independent experiments in which a total 900 virus particles were analyzed in 30 cells. Bars, 5 µm. **, P < 0.01; ****, P < 0.0001.

Previous observations have demonstrated that dynamin II (dyn II), which is recruited by enteropathogenic *Escherichia coli* (EPEC)–inducing actin pedestals (Unsworth et al., 2007), interacts with the third SH3 domain of Nck (Wunderlich et al., 1999). Given the parallels between EPEC pedestals and vaccinia-induced actin tails (Welch and Way, 2013), we examined if dynamin might be responsible for the displacement of septins from CEV. We found that dyn II is recruited to 12.53 ± 0.5% of CEV in WR infected HeLa cells (Fig. 8 A). In contrast, in A36-YdF virus–infected cells, dyn II is only associated with 3.1 ± 0.2% of CEV (Fig. 8 A). At 8 h after infection, 12.41 ± 1.91% and 30.29 ± 3.54% of CEV in HeLa cells recruit dynamin and Nck, respectively, whereas 9.0 ± 1.96% of viruses have both proteins (Fig. 8 B). RNAi-mediated depletion of SEPT7 results in a significant increase in the number of CEVs with dyn II in WR-infected HeLa cells (Fig. 8 C). To test if dyn II is recruited to CEV downstream of Nck, we infected Nck^−/−^ MEFs. In the absence of Nck, the number of CEVs recruiting dynamin is equally low in both WR- and A36-YdF–infected cells. In Nck^+/+^ MEFs, as seen in HeLa cells, significantly fewer CEVs recruit dynamin in A36-YdF– but not WR-infected cells (Fig. 8 D). Furthermore, point mutation of the third (Δ3) but not the first and second (Δ1+2) SH3 domain of Nck reduces dynamin recruitment to CEV in WR-infected Nck^−/−^ MEFs stably expressing GFP-tagged Nck mutants (Fig. 8 E).

**Figure 9.**
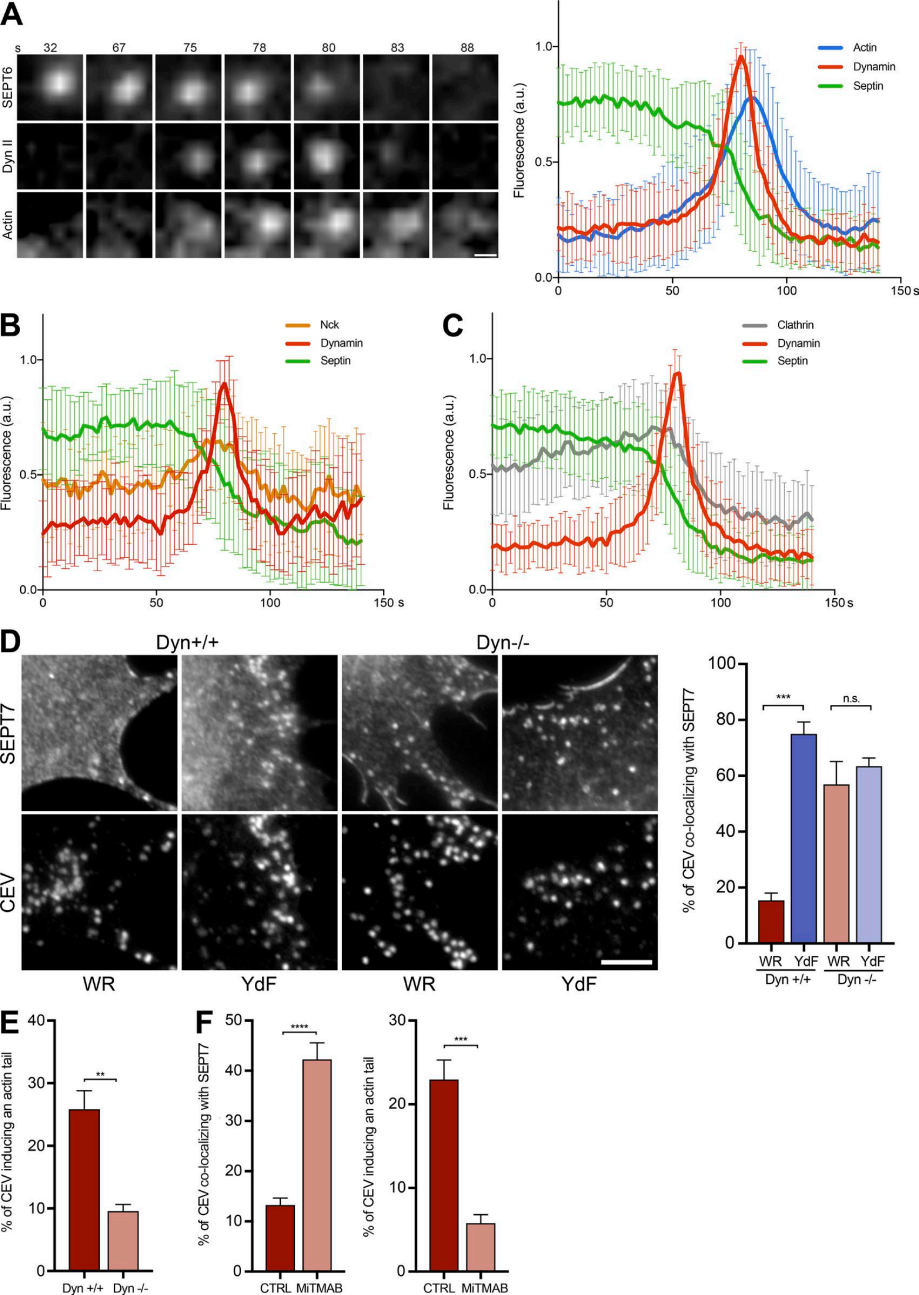
**Dynamin promotes loss of septin from CEV. (A)** Video stills of the association of GFP-SEPT6, mCherry-dynamin (Dyn) II, and LifeAct-iRFP (Actin) with CEV in WR-infected HeLa cells at the indicated time. See Video 6. The graph shows the mean normalized fluorescence intensity of each protein on the virus over time. Loss of septin coincides with the recruitment of dynamin and actin polymerization before virus movement. **(B)** The graph shows the mean normalized fluorescence intensity of GFP-SEPT6, iRFP–dyn II, and RFP-Nck associated with CEV in HeLa cells infected with WR. The time in seconds is shown above the stills. See Fig. S4 B and Video 7. **(C)** The graph shows the mean normalized fluorescence intensity of GFP-SEPT6, mCherry-clathrin light chain (Clathrin), and iRFP–dyn II on CEV in WR-infected HeLa cells over time. See Fig. S4 C and Video 8. **(D)** Immunofluorescence images showing the recruitment of SEPT7 to CEV in dynamin parental (Dyn +/+) and knockout (Dyn −/−) cells infected with WR and the YdF virus. Bars, 5 µm. The graph shows the percentage CEV in WR- and YdF-infected cells recruiting SEPT7 in the presence (+/+) or absence (−/−) of dynamin at 14 h after infection. **(E)** The graph shows the percentage of CEVs inducing actin tails in WR-infected cells in the presence (+/+) or absence (−/−) of dynamin. **(F)** Quantification of the percentage of CEV recruiting SEPT7 and inducing actin tails in WR-infected HeLa cells treated with the dynamin inhibitor MiTMAB. In the graphs in A, B, and C, the error bars represent SD from three independent experiments in which a total of >70 virus particles were analyzed. In the graphs in D and E, the error bars represent SEM from three independent experiments in which >900 CEVs were analyzed across 30 cells. In the graphs in F, >1,500 virus particles from five independent experiments were analyzed across 50 cells. Bars: (A) 500 nm; (D) 5 µm. **, P < 0.01; ***, P < 0.001; ****, P < 0.0001.

The relatively low numbers of CEV with dyn II suggests that its recruitment is likely to be transient. We performed quantitative live cell imaging to examine if this is the case and to determine whether recruitment of dynamin correlates with the loss of septins from CEV. We found that as dyn II and actin accumulate on CEV, a corresponding reduction in SEPT6 is observed (Fig. 9 A and Video 6). Nck also accumulates as septin is lost, reaching its maximum intensity just before that of dynamin (Fig. 9 B, Fig. S4 B, and Video 7). In contrast to septin, clathrin remains associated with the virus until maximal dyn II accumulation before virus movement (Fig. 9 C, Fig. S4 C, and Video 8).

To confirm that dynamin recruitment is responsible for displacing septins, we took advantage of conditional mouse knockout fibroblasts in which tamoxifen treatment leads to loss of all three dynamin isoforms (Ferguson et al., 2009; Park et al., 2013). We found that in the absence of dynamin, the number of CEVs recruiting SEPT7 is equally high for WR and the A36-YdF virus (Fig. 9 D and Fig. S4 D). This contrasts with the situation in MEFs that have not been treated with tamoxifen, where SEPT7 association with CEV remains low in WR-infected cells (WR 15.5 ± 2.5%, YdF 75.1± 4.2%; Fig. 9 D). Loss of dynamin also results in a significant drop in the number of CEVs inducing actin tails (Dyn^+/+^ 25.9 ± 3.0% and Dyn^−/−^ 9.6 ± 1.1%; Fig. 9 E). Additionally, treatment of WR-infected HeLa cells with the dynamin inhibitor MiTMAB significantly increases the number of CEVs with associated SEPT7 and reduces the number of actin tails (Fig. 9 F). Taken together, our observations clearly demonstrate that Nck-mediated recruitment of dyn II promotes the loss of septins from CEV before actin tail formation.

### Dynamin promotes septin loss in a formin-dependent fashion

**Figure 10.**
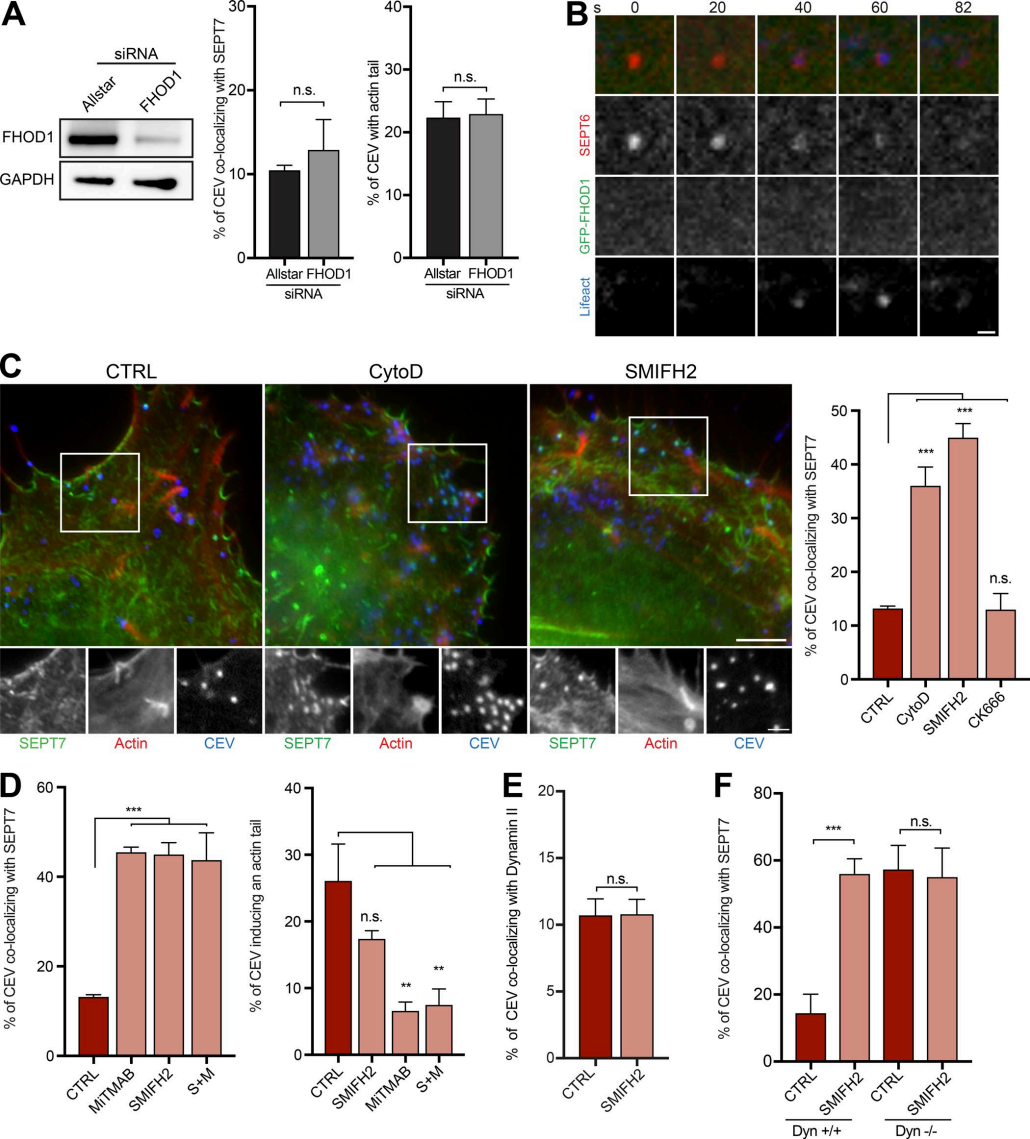
**Formin-mediated actin polymerization displaces septin from CEV. (A)** Immunoblot analysis confirming that FHOD1 siRNA treatment of HeLa cells for 72 h leads to loss of FHOD1. The graphs show the percentage of CEV recruiting SEPT7 and inducing actin tails in the presence (Allstar) or absence of FHOD1. **(B)** Representative images panels from live cell imaging showing that GFP-FHOD1 is not recruited to CEV when septins are displaced from the virus. The time in seconds is indicated. Bar, 1 µm. See Video 9. **(C)** Images showing the association of SEPT7 with CEV in WR-infected HeLa cells treated with cytochalasin D to block actin polymerization or SMIFH2 to inhibit formins. The graph shows quantification of septin recruitment during the indicated drug treatments. Bars: (main images) 5 µm; (insets) 2 µm. **(D)** Quantification of the impact of inhibiting formins (SMIFH2), dynamin (MiTMAB) or both proteins on the % of CEV recruiting SEPT7 and inducing actin tails. **(E)** The graph shows the effect of inhibiting formins with SMIFH2 on the percentage of CEV recruiting dyn II. **(F)** Quantification of the percentage of CEV recruiting SEPT7 in the presence (+/+) or absence (−/−) of dynamin with and without (CTRL) SMIFH2 treatment. All error bars represent SEM from three independent experiments in which >900 CEVs were analyzed across 30 cells. **, P < 0.01; ***, P < 0.001.

It is striking that there is a concomitant accumulation of dynamin and actin as septin is progressively lost before virus movement (Fig. 9 A). This suggests that actin polymerization may participate in septin displacement; however, inhibition of the Arp2/3 complex has no impact on the number of CEVs recruiting septin (Fig. 6 B). Previous observations demonstrated that the formin FHOD1 is recruited to vaccinia-induced actin tails, and its presence increases their number (Alvarez and Agaisse, 2013). We therefore tested if FHOD1 is responsible for nucleating the actin that appears as septins are lost from the virus. We found that loss of FHOD1 had no impact on the number of CEVs recruiting septin or inducing actin tails (Fig. 10 A). This suggests that it is not responsible for nucleating the actin that displaces septin from CEV. Consistent with this, we do not see recruitment of GFP-FHOD1 to the virus when septin is displaced from CEV (Fig. 10 B and Video 9). GFP-FHOD1 is, however, recruited to actin tails (Fig. S5, A and B; and Video 10).

To examine if another formin is responsible for nucleating the actin that displaces septins, we treated WR-infected HeLa cells with the formin inhibitor SMIFH2 (Rizvi et al., 2009). We found that treatment of WR-infected HeLa cells with 100 µM SMIFH2 for 30 min induced cytotoxic cell rounding and blocked actin tail assembly (Fig. S5 C). In contrast, exposing cells to 20 µM SMIFH2 for the same time had no apparent side effects and did not abolish actin tail formation (Fig. S5 C). However, under these conditions, the number of CEVs colocalizing with septins increased to a similar extent as seen when inhibiting actin polymerization with cytochalasin D (Fig. 10 C). The simultaneous inhibition of dynamin (MiTMAB) and formins (SMIFH2) did not increase the number of CEVs with septins above that seen with the individual inhibitors (Fig. 10 D). This suggests that during septin displacement, dynamin and formins do not act independently of each other. Treatment of infected cells with either MiTMAB or SMIFH2 reduces the number of CEVs inducing actin tails (Fig. 10 D). Again, combining both MiTMAB and SMIFH2 does not further reduce actin tail numbers (Fig. 10 D). SMIFH2 alone had no impact on the number of CEVs recruiting dynamin in WR-infected HeLa cells (Fig. 10 E). However, the increase in SEPT7 recruitment seen in the presence of SMIFH2 is dynamin-dependent (Fig. 10 F). Taken together, our data suggest that dynamin, together with formin-mediated actin polymerization, displaces septins from CEV to facilitate actin tail formation.

## Discussion

Previous genome-wide RNAi-based screens found that loss of septins enhances vaccinia replication and/or viral spread (Sivan et al., 2013; Beard et al., 2014). We have now found that septins exert their antiviral effect by forming cage-like structures around CEVs to suppress the release of virions from infected cells. Our observations demonstrate that the ability of septins to limit the spread of infection is not only restricted to bacterial pathogens such as *S. flexneri* (Mostowy et al., 2010; Sirianni et al., 2016). We found that septins are transiently recruited to CEV independently of clathrin before actin tail formation. Septins are not required for actin tail formation, but their loss increases the number of CEVs stimulating actin polymerization. This is reminiscent of the response of septin loss during *S. flexneri* infection (Mostowy et al., 2010). Septin-mediated suppression of actin tail formation will clearly limit the cell-to-cell spread of vaccinia. However, the antiviral action of septins goes beyond mere suppression of actin tail formation, as the release and spread of the A36-YdF virus from infected cells is also significantly reduced. The prominent recruitment of septins to CEV combined with its ability to “cage” the virus may explain the close association of the A36-YdF virus within plasma membrane invaginations that were previously described (Horsington et al., 2013).

The 13 human septins, which are grouped into four different homology groups (SEPT2, SEPT3, SEPT6, and SEPT7), play unique and/or redundant roles in septin filament assembly (Saarikangas and Barral, 2011; Mostowy and Cossart, 2012; Neubauer and Zieger, 2017). We found that CEVs recruit septins from each homology group, SEPT2 (SEPT2 group), SEPT6/SEPT11 (SEPT6 group), SEPT7 (SEPT7 group), and SEPT9 (SEPT3 group). Moreover, in agreement with the previous genome-wide RNAi-based screens, we found that SEPT2, SEPT7, and SEPT11 are antiviral, with their loss increasing the spread of vaccinia. Beard et al. (2014) found that SEPT1 (SEPT2 group) and SEPT9 are also antiviral. Taken together, this suggests that multiple septins are capable of suppressing the release of CEV from infected cells. It is therefore likely that the antiviral impact of septins on vaccinia will occur in most cell types regardless of their septin expression profiles.

Our initial observations from fixed images and live cell imaging suggested that septin loss from CEV is triggered by Arp2/3-dependent actin tail formation. However, this is not the case as neither the chemical inhibition of the Arp2/3 complex nor the absence of N-WASP results in greater numbers of CEVs with septins during WR infection. In contrast, we found that it is the phosphorylation of tyrosine 112 of A36 and the subsequent recruitment of Nck that trigger the sequence of events leading to septin displacement. Crucially, the recruitment of dynamin by the third SH3 domain of Nck is required to displace septins from the virus.

Dynamin is a large GTPase that regulates membrane fission during clathrin-mediated endocytosis and the actin cytoskeleton during a range of cellular processes (Ferguson and De Camilli, 2012; Menon and Schafer, 2013; Sever et al., 2013). Quantitative live cell imaging reveals that septin is progressively lost as dynamin and actin accumulate on the virus. This contrasts with the behavior of clathrin, which remains associated with CEV until dynamin accumulation reaches its peak and the virus undergoes actin-based motility. Interestingly, the timing of dynamin, clathrin, and actin recruitment to the CEV is similar to what has been described during clathrin-mediated endocytosis (Taylor et al., 2011). The transient recruitment of dyn II to CEV before actin tail formation may explain why it was previously missed (Scaplehorn et al., 2002; Unsworth et al., 2007). This is in contrast to infection by the bacterial pathogen EPEC, where dyn II is readily detected on actin pedestals (Unsworth et al., 2007; Humphries and Way, 2013). Interestingly, as we have found with vaccinia, dyn II recruitment to EPEC depends on Nck, but is independent of the Arp2/3 complex (Unsworth et al., 2007). Moreover, loss of dyn II leads to a reduction in both EPEC- and vaccinia-induced actin structures (Fig. 9 E; Unsworth et al., 2007). Consistent with our current observations, the loss of SEPT9 significantly decreases EPEC adherence to HeLa cells (Scholz et al., 2015). It is likely that adherent EPEC will also recruit septin before actin pedestal formation.

Chemical inhibition or the absence of dynamin increases the number of CEV with SEPT7 and reduces their capacity to induce actin tails. In contrast, loss of septins leads to more CEV with dyn II and increased actin tail formation. This reciprocal relationship clearly demonstrates that dyn II overcomes the ability of septins to suppress vaccinia actin tail formation. Moreover, live cell imaging reveals that septins are rapidly recruited back to CEV if actin-based motility ceases (Fig. S3 C and Video 3). The dissociation of septins from the virus is marked by a concomitant accumulation of dynamin and actin. The global inhibition of actin polymerization with cytochalasin D increases the number of CEV with SEPT7, whereas specific inhibition of Arp2/3-dependent actin assembly with CK666 has no impact on septin recruitment. This suggests that the actin accumulation seen during septin loss is more likely a result of other actin nucleators such as formins or the direct interaction of dynamin with actin, rather than via N-WASP/cortactin or its other binding partners (Gu et al., 2010; Ferguson and De Camilli, 2012; Menon and Schafer, 2013; Sever et al., 2013).

Previous observations have demonstrated that the formin FHOD1 promotes vaccinia virus spread in confluent cell monolayers as well as actin tail formation (Alvarez and Agaisse, 2013). The same study also found that FHOD1 is localized throughout actin tails in an N-WASP–dependent fashion. Given these observations, we examined whether FHOD1 was responsible for the actin burst observed when septins are lost from CEV. We found that the loss of FHOD1 had no impact on the number of CEVs recruiting SEPT7. More surprisingly, siRNA-mediated depletion of FHOD1 had no effect on the number of CEVs inducing actin tails. GFP-FHOD1, however, is recruited to actin tails but not to CEV at the point when septins are lost. We are unable to explain why our observations on the role of FHOD1 in actin tail formation are different from those of Alvarez and Agaisse (2013). Notwithstanding this, there is a fundamental difference in the level at which FHOD1 (formin) is working between the two studies. The data of Alvarez and Agaisse (2013) demonstrate that FHOD1 is recruited downstream of N-WASP. In our case, the actin nucleating activity of the “formin” displacing septin is independent of N-WASP, as in WR-infected N-WASP^−/−^ cells, septin recruitment is as low as control N-WASP^+/+^ cells (Fig. 6 C). The recruitment of GFP-FHOD1 to actin tails downstream of N-WASP is consistent with its ability to cap and bundle actin filaments (Schönichen et al., 2013; Patel et al., 2018). Our data question the involvement of FHOD1 in vaccinia actin tail formation, but clearly demonstrate that it is not involved in displacing septins from CEV.

Our evidence that formin-dependent actin polymerization promotes septin loss is based on the use of the chemical inhibitor SMIFH2 (Rizvi et al., 2009). In vitro, SMIFH2 has a half-maximal inhibition of mDia-mediated actin assembly at ∼15.0 µM. Treating NIH3T3 fibroblasts with a range of SMIFH2 concentrations reveals that the inhibitor induces cytotoxicity (cell rounding and blebbing) at an IC50 of 28.0 µM (Rizvi et al., 2009). Consistent with this, we found that treating HeLa cells infected with vaccinia for 8 h with 100 µM SMIFH2 for 30 min induces cell rounding and suppresses vaccinia-induced actin polymerization. This is in agreement with Borinskaya et al. (2016), who also found that 100 µM SMIFH2 suppresses vaccinia actin tail formation. To avoid cell toxicity and potential side effects, we used SMIFH2 at a final concentration of 20 µM, which is in line with other studies (Isogai et al., 2015). At this concentration, there are no apparent cytotoxic effects, but the number of CEVs recruiting septins increases significantly, as seen with cytochalsin D but not the Arp2/3 complex inhibitor CK666. The combined inhibition of dynamin and formin had no additive effect on septin recruitment, indicating that both proteins are required to alleviate the antiviral effect of septins. The task ahead is to identify the formin responsible for displacing septin from CEV and confirm whether it is acting downstream of dynamin.

Our previous observations demonstrate that after IEV fuse with the plasma membrane, CEV transiently recruit clathrin before actin tail formation (Humphries et al., 2012). Our live cell imaging suggests that in contrast to septins, the accumulation of dynamin does not appear to impact clathrin, which is only lost when the virus undergoes actin-based motility. We also found that septins are always recruited before the appearance of clathrin on the virus. However, septins and clathrin do not influence the recruitment of each other. Both proteins assemble into supermolecular structures, raising the intriguing question of how septins and clathrin are actually arranged on the virus. In addition, our FRAP analysis reveals that just over half the septins “caging” CEV are able to undergo turnover. A similar result is observed for YFP-SEPT2 in septin filaments and rings in normal rat kidney epithelial cells and MDCK cells (Schmidt and Nichols, 2004; Hu et al., 2008). The presence of both a dynamic and stable population suggests that the septin assemblies may exist in two different conformations. In light of this, it is interesting that in budding yeast, septins are also present in two different filaments assemblies in the mother-bud junction (Ong et al., 2014). A complete understanding of how both septins and clathrin are organized, their relationship to dynamin, and their subsequent actin tail assembly will require detailed ultrastructural analysis of CEV on the plasma membrane.

In summary, we have uncovered a new function for septins and their regulation by dynamin. Our study represents the first example where septins play an important, albeit an inhibitory, role during virus spread. It will be interesting to see whether septins also suppress the release of other viruses, such as herpes virus, when they fuse with the plasma membrane. It is also highly likely that septins will have additional functions during other viral infections given their diverse interactions and cellular functions.

## Materials and methods

### Antibodies, drugs, expression clones, and cells

The following primary antibodies were used for immunofluorescence and immunoblotting: A36 (Röttger et al., 1999), AP-2 (2730; Abcam), Clathrin Heavy Chain (21679; Abcam), B5 (Hiller and Weber, 1985; the B5 antibody was labeled with the Molecular Probes Alexa Fluor 488 Monoclonal Antibody Labeling kit [Invitrogen] to generate the Alexa Fluor 488-B5 antibody [Humphries et al., 2012]), dyn II for immunofluorescence (Henley et al., 1998; provided by M. McNiven, Mayo Clinic, Rochester, NY) and Western (A303-513A; Bethyl), FHOD1 (A304-825A; Bethyl), GAPDH (ab8245; Abcam), SEPT2 (60075–1-Ig; ProteinTech), SEPT7 (18991; IBL), SEPT9 (10769–1-AP; ProteinTech) for immunofluorescence and SEPT9 (A302-353A; Bethyl) for Western, and SEPT11 (A304-176A; Bethyl). Secondary antibodies and fluorescently labeled phalloidin were obtained from Invitrogen. CK666 was obtained from Sigma-Aldrich (SML0006) and added to a final concentration of 100 µM after 7 h of infection for 1 h before fixation. PP1 was obtained from Cayman Chemical (14244) and added to a final concentration of 12.5 µM after 4 h of infection for an additional 4 h before fixation. MiTMAB was obtained from Abcam (ab120466) and added to a final concentration of 30 µM after 7.5 h of infection, and cells were fixed 30 min later. SMIFH2 was obtained from Torcis (4401) and added to a final concentration of 20 µM after 7.5 h of infection, and cells were fixed 30 min later. CK666, PP1, MiTMAB, and SMIFH2 were dissolved in DMSO.

The pE/L expression vector encoding mCherry-tagged clathrin light chain was described before (Humphries et al., 2012). The pE/L RFP-Nck1 plasmid was generated by inserting human Nck1 into the NotI and EcoRI sites of the pE/L vector (Frischknecht et al., 1999; Arakawa et al., 2007b). The pE/L expression vector encoding GFP-FHOD1 was generated by cloning human FHOD1, provided by A. Alberts (Van Andel Institute, Grand Rapids, MI), into the EcoRI and NotI sites of pE/L GFP vector (Frischknecht et al., 1999). HeLa, BS-C-I, and A549 cell lines were maintained in MEM supplemented with 10% FBS, 100 U/ml penicillin, and 100 µg/ml streptomycin at 37°C and 5% CO_2_. HeLa and A549 cells are originally from ATCC. The Nck^−/−^ MEFs (Bladt et al., 2003), N-WASP^−/−^ MEFs (Snapper et al., 2001), and Dyn^−/−^ fibroblasts (Ferguson et al., 2009; Park et al., 2013) were provided by the late T. Pawson (Samuel Lunenfeld Research Institute, Toronto, Canada), S. Snapper (Harvard Medical School, Boston, MA), and P. De Camilli (Yale School of Medicine, New Haven, CT), respectively, and maintained in Dulbecco**’**s modified Eagle**’**s medium supplemented with 10% FBS, 100 U/ml penicillin, and 100 µg/ml streptomycin. Dynamin was depleted by treating from Dyn^−/−^ fibroblasts with tamoxifen as previously described (Ferguson et al., 2009; Park et al., 2013).

All cell lines used in this study have been negatively tested for mycoplasma. Nck^−/−^ MEFs stably expressing GFP**-**Nck1 variants have been previously described (Donnelly et al., 2013). HeLa cells stably expressing GFP-SEPT6 (Sirianni et al., 2016) were infected with the lentiviral expression vector pLVX–Lifeact–iRFP670 (Snetkov et al., 2016) to generate cells stably expressing both GFP-SEPT6 and Lifeact–iRFP670. HeLa cells stably expressing GFP-SEPT6 were also infected with the lentiviral expression vector pLVX–iRFP670- dyn II to generate cells stably expressing both GFP-SEPT6 and iRFP670–dyn II. The lentiviral expression vector pLVX–iRFP670–dyn II was generated by cloning iRFP670 (Shcherbakova and Verkhusha, 2013) into the EcoRI and NotI sites of pLVX-IRES hygro vector (Clonetech). Rat dyn II (Cao et al., 1998) was subsequently inserted into the NotI site of the iRFP670 pLVX-IRES hygro vector. In addition, the HeLa cells stably expressing GFP-SEPT6 and Lifeact–iRFP670 were also infected with the lentiviral expression vector pLVX–mCherry–dyn II to generate cells stably expressing all three proteins. The lentiviral expression vector pLVX**–**mCherry–dyn II was generated by cloning rat dyn II into the NotI and EcoRI sites of the pLVX mCherry puro vector (Dodding et al., 2011). All lentiviruses were prepared and used to infect HeLa cells as described previously (Abella et al., 2016). HeLa cells stably expressing the desired proteins were selected and maintained in culture medium containing 50 µg/ml hygromycin B and/or 1 µg/ml puromycin.

### Infection, RNAi treatment, and immunofluorescence analysis

Cells were infected with vaccinia virus 24 h after plating on fibronectin at an MOI of 5 and fixed at 8 h (HeLa), 10 h (N-WASP MEFs), 14 h (Dyn fibroblasts), or 20 h (Nck MEFs) after infection to be processed for immunofluorescence as previously described (Arakawa et al., 2007a,b). Extracellular CEVs attached to the plasma membrane were detected using the B5 antibody before cell permeabilization with detergent. Fixed cells were imaged with a 63×/1.4 Plan Achromat objective on Zeiss Axioplan2 microscope equipped with a Photometrics Cool Snap HQ cooled charge-coupled device camera. The system was controlled with MetaMorph 7.8.13.0 software.

High-resolution confocal imaging of fixed cells was performed using a 63×/1.4 C-Plan Apo oil immersion lens on a Zeiss LSM 880 microscope in Airyscan mode. Image acquisition and processing of Airyscan datasets was performed with Zeiss Zen software. Where required, cells were transfected with 20 µM siRNA as per the Hiperfect fast-forward protocol (QIAGEN). For plaque assays, confluent A549 cells were transfected twice 48 h apart with control or SEPT7 siRNA before infection. HeLa cells were transfected with siRNA for 72 h before infection for 8 h followed by processing for immunofluorescence or immunoblot analysis. siRNA oligonucleotides targeting SEPT2 (D-010614-03/05/20/21), SEPT7 (D-011607-01/02/04/18), SEPT9 (D-006373-02/03/05/6), SEPT11 (D-020249-01to04), and FHOD1 (D-013709-01/02/03/04) were obtained from Dharmacon, and Allstar control was obtained from QIAGEN (SI03650318).

### Live cell imaging

HeLa cells were plated onto Matek dishes coated with fibronectin 24 h before infection. Cells were infected at an MOI of 5 and imaged at 37°C in phenol red free MEM containing 40 mM Hepes from 7 h after infection on a Zeiss Axio Observer microscope equipped with a Plan Achromat 63×/1.4 Ph3 M27 oil lens, an Evolve 512 camera, and a Yokagawa CSUX spinning disk under the control of Slidebook 6.0 (Intelligent Imaging). Videos were acquired either every 3 s to measure arrival of septin and clathrin before actin tail formation or actin tail duration, or every 2 s for all the other videos.

### Plaque assays and virus release assays

Plaque assays were performed in A549 cells for 72 h before fixation, and subsequently visualized using immunofluorescent staining of B5 as previously described (Humphries et al., 2012). Plaque diameter was quantified manually using Fiji. To quantify release of extracellular enveloped virus, 12-well dishes were seeded with A549 cells and infected at an MOI of 5 in serum-free MEM. Supernatants were collected at 18 h after infection and used to determine virus release (plaque-forming units/milliliter). The level of intracellular virus was determined by trypsinizing the remaining cells, which after pelleting were resuspended in 10 mM Tris, pH 9.0, and 2 mM MgCl_2_ and lysed by three freeze-thawing cycles. In both cases, the infectious virus was titrated by infecting confluent BS-C-1 cells with 10-fold serial dilutions. At 48 h after infection, BS-C-1 cells were stained with crystal violet, and the number infectious virus was quantified by counting plaques. All release assays were performed in duplicate and over three independent experiments.

### Quantification and figure preparation

Quantification of the number CEVs inducing an actin tail was determined by analyzing >900 CEVs in 10 randomly selected cells in three independent experiments. Actin tail length was measured using Fiji for 370 actin tails in 10 individual cells in three independent experiments. Analysis of actin tail duration and protein arrival before actin polymerization was performed in Fiji. For automated quantification of protein intensity levels on CEV, images were opened in Fiji, and an area of roughly 2.5 µm^2^ around the virus was selected and cropped. Cropped image stacks were loaded into MATLAB (R2017A), where the normalized fluorescence intensities were determined. All intensity values were aligned temporally with respect to the peak dynamin intensity, determined by the Savitzky-Golay filtered data. The mean and SD of all the unfiltered intensity values were plotted using Prism. Colocalization analysis in fixed cells was quantified for >900 CEVs in 10 randomly selected cells in three independent experiments. All data are presented as means ± SEM and were analyzed by Student’s *t* test when comparing two conditions and one-way analysis of variance with Tukey**’**s multiple comparison test when comparing more than two conditions, using Prism 7 (GraphPad Software). All figures and graphs were prepared using Prism and Illustrator software.

### Online supplemental material

Fig. S1, related to Fig. 1, shows the impact of siRNA-mediated loss of septins on viral cell-to-cell spread. Fig. S2, related to Fig. 2, shows the impact of siRNA-mediated loss of septins on actin tails. Fig. S3, related to Figs. 3 and 4, shows CEV recruit different septins and dynamics of GFP-SEPT6 on CEV. Fig. S4, related to Figs. 5 and 9, shows GFP-SEPT6 dynamics on CEV with respect to actin, clathrin, dynamin, and Nck1. Fig. S5, related to Fig. 10, shows the impact of formin inhibition with SMIFH2 on actin tail formation and the recruitment of GFP-FHOD1 to actin tails. Video 1, related to Fig. 3 B, shows the recruitment of GFP-SEPT6 to CEV. Video 2, related to Fig. 4 B, shows the recruitment of GFP-SEPT6 to CEV and its subsequent loss when the virus induces actin polymerization. Video 3, related to Fig. S3 C, shows GFP-SEPT6 is recruited to CEV when they cease actin-based motility. Video 4, related to Fig. 5 B, shows the recruitment of SEPT6 and clathrin to CEV and their subsequent loss when actin polymerization starts. Video 5, related to Fig. S4 A, shows SEPT6 can be recruited to CEV in the absence of clathrin. Video 6, related to Fig. 9 A, shows SEPT6 is lost from virus as dynamin and actin accumulate. Video 7, related to Fig. S4 B, shows SEPT6 is lost from virus as dynamin and Nck accumulate. Video 8, related to Fig. S4 C, shows SEPT6 and clathrin loss as dynamin accumulates on the virus. Video 9, related to Fig. 10 B, shows GFP-FHOD1 is not recruited to CEV when SEPT6 is lost as actin accumulates. Video 10, related to Fig. S5 A, shows the recruitment of GFP-FHOD1 to vaccinia-induced actin tails.

## Supplementary Material

Supplemental Materials (PDF)

Video 1

Video 2

Video 3

Video 4

Video 5

Video 6

Video 7

Video 8

Video 9

Video 10
