# Supplementary material for: Septins suppress the release of vaccinia virus from infected cells
Source: J Cell Biol. 2018 Aug 6;217(8):2911–29. doi: 10.1083/jcb.201708091 (PMC6080921; doi:10.1083/jcb.201708091)
Supplement: Supplemental Materials (PDF) [file JCB_201708091_sm.pdf]

JCB

Supplemental material

Pfanzelter et al., https://doi.org/10.1083/jcb.201708091

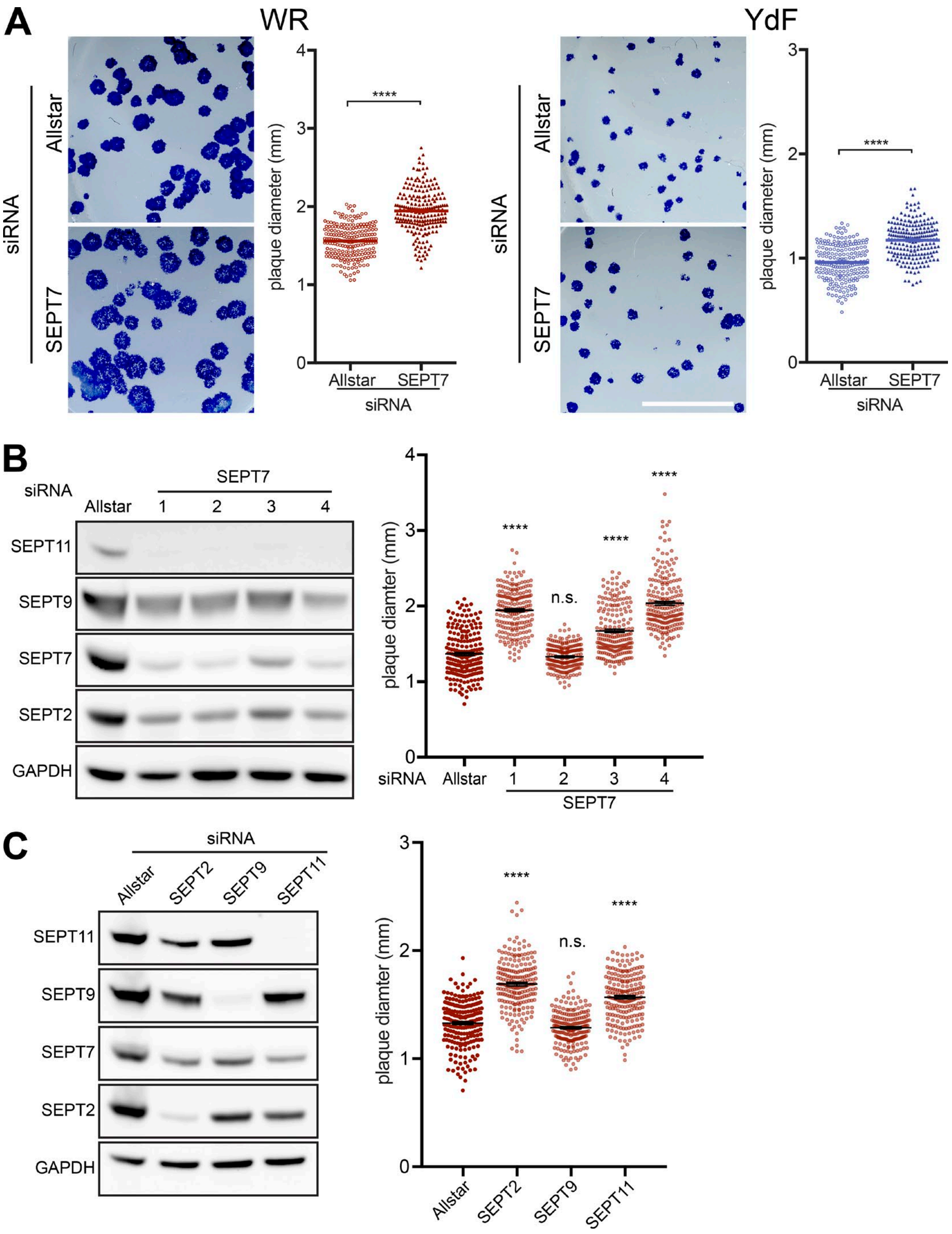

Figure S1. **Septin reduces virus spread. (A)** Images of plaques formed on A549 cell monolayers treated with control (Allstar) or SEPT7 siRNA for 72 h under semisolid overlay after 3 d of infection with WR strain or the YdF virus. Bar, 1 cm. **(B)** Immunoblot analysis confirming that treatment of A549 cells for 72 h with individual deconvoluted SEPT7 siRNA from the pool in Fig. 1 reduces expression SEPT7 as well as SEPT2, SEPT9, and SEPT11. The graph shows the quantification of plaque size (n = 210) in the treated cells with error bars representing the SEM from three independent experiments for the indicated siRNA. **(C)** Immunoblot analysis confirming that treatment of A549 cells for 72 h with SEPT2, SEPT9, or SEPT11 siRNA reduces expression of the corresponding protein. The graph shows the quantification of plaque size (n = 210) in the treated cells with error bars representing the SEM from three independent experiments for the indicated siRNA. ****, $P < 0.0001$.

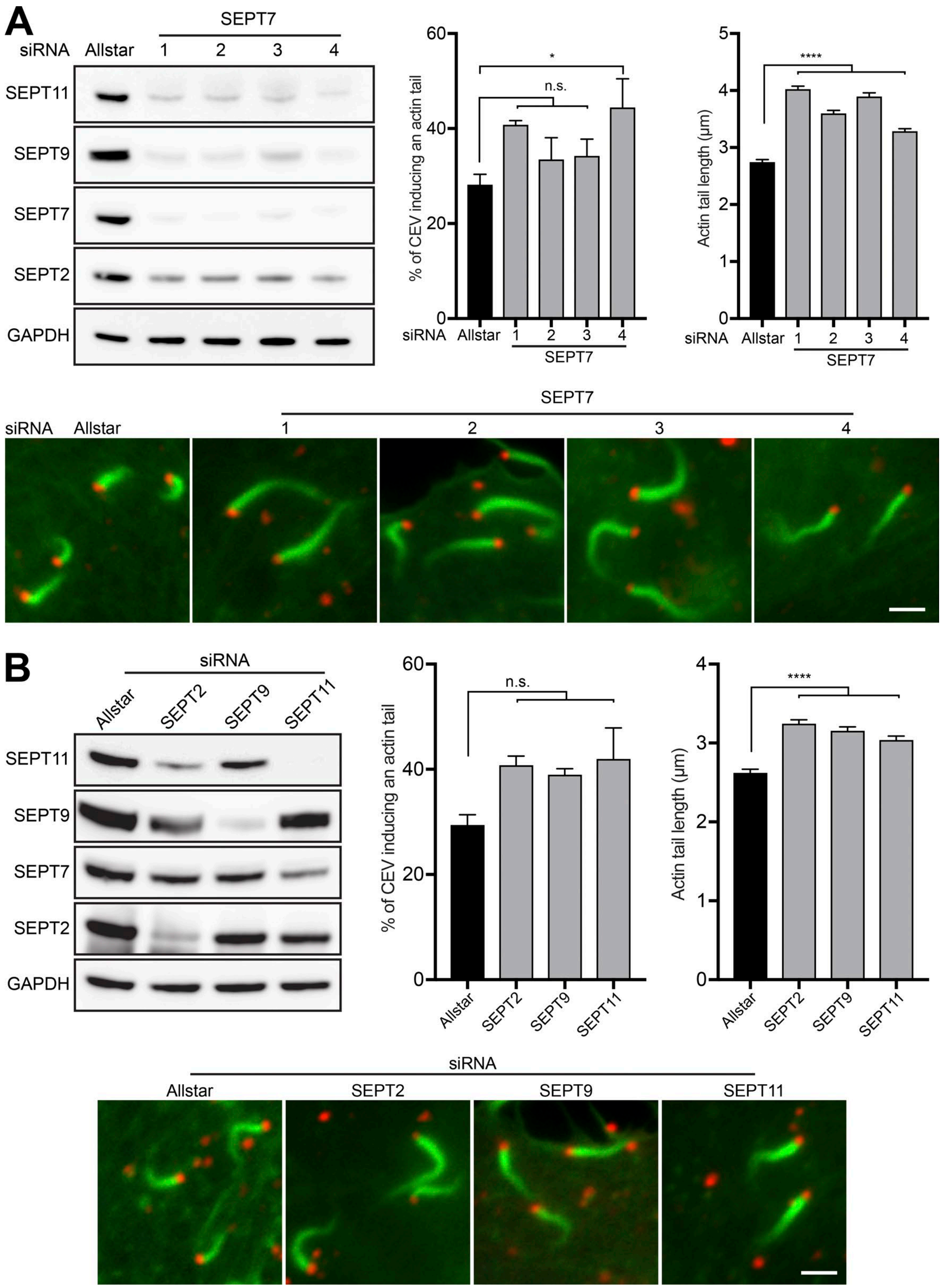

Figure S2. **Loss of septin promotes actin tail formation. (A)** Immunoblot analysis confirming that treatment of HeLa cells for 72 h with the indicated SEPT7 siRNA reduces expression SEPT7 as well as SEPT2, SEPT9, and SEPT11. The graph shows the quantification of the number of CEVs inducing actin tails and their length in the treated cells. Representative images of actin tails (green) induced by WR (red) in HeLa cells treated with the different SEPT7 siRNAs are also shown. **(B)** Immunoblot analysis confirming that treatment of HeLa cells for 72 h with SEPT2, SEPT9, or SEPT11 siRNA reduces expression of the corresponding protein. The graph shows the quantification of the number of CEVs inducing actin tails and their length in the treated cells. Representative images of actin tails (green) induced by WR (red) in HeLa cells treated with the different siRNA are also shown. Error bars represent SEM from three independent experiments in which a total of 30 cells were analyzed for actin tail number and 300 tails were measured for length. Bar, 2 µm. *, $P < 0.05$; ****, $P < 0.0001$.

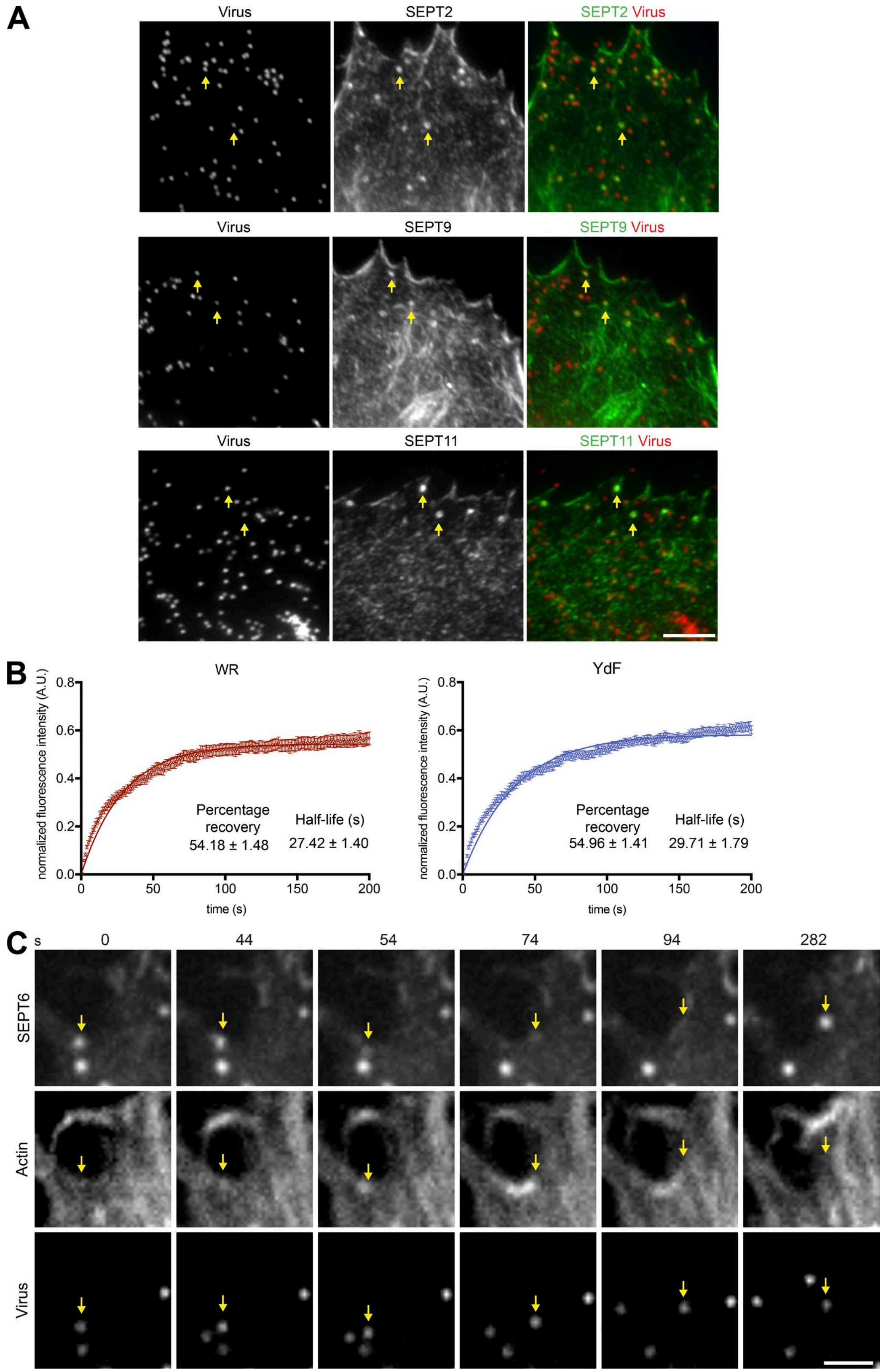

Figure S3. **Septin competes with actin tail formation. (A)** Representative immunofluorescence images showing the recruitment of endogenous SEPT2, SEPT9, and SEPT11 to virus particles (yellow arrows) in HeLa cells infected with WR expressing A3-RFP. Bar, 5 µm. **(B)** Quantification of the normalized fluorescent intensity of GFP–SEPT6 on CEV in WR- and YdF-infected cells during recovery after photobleaching. In each case, a total of >35 viruses was bleached in three independent experiments, and the error bars represent SEM. **(C)** Video stills taken from live cell imaging of HeLa cells expressing GFP-SEPT6 and Lifeact-iRFP (Actin) after infection with WR expressing A3-RFP (Virus) for 8 h. Septin is lost upon actin tail formation but returns when actin polymerization ceases. See Video 3. Bar, 3 µm.

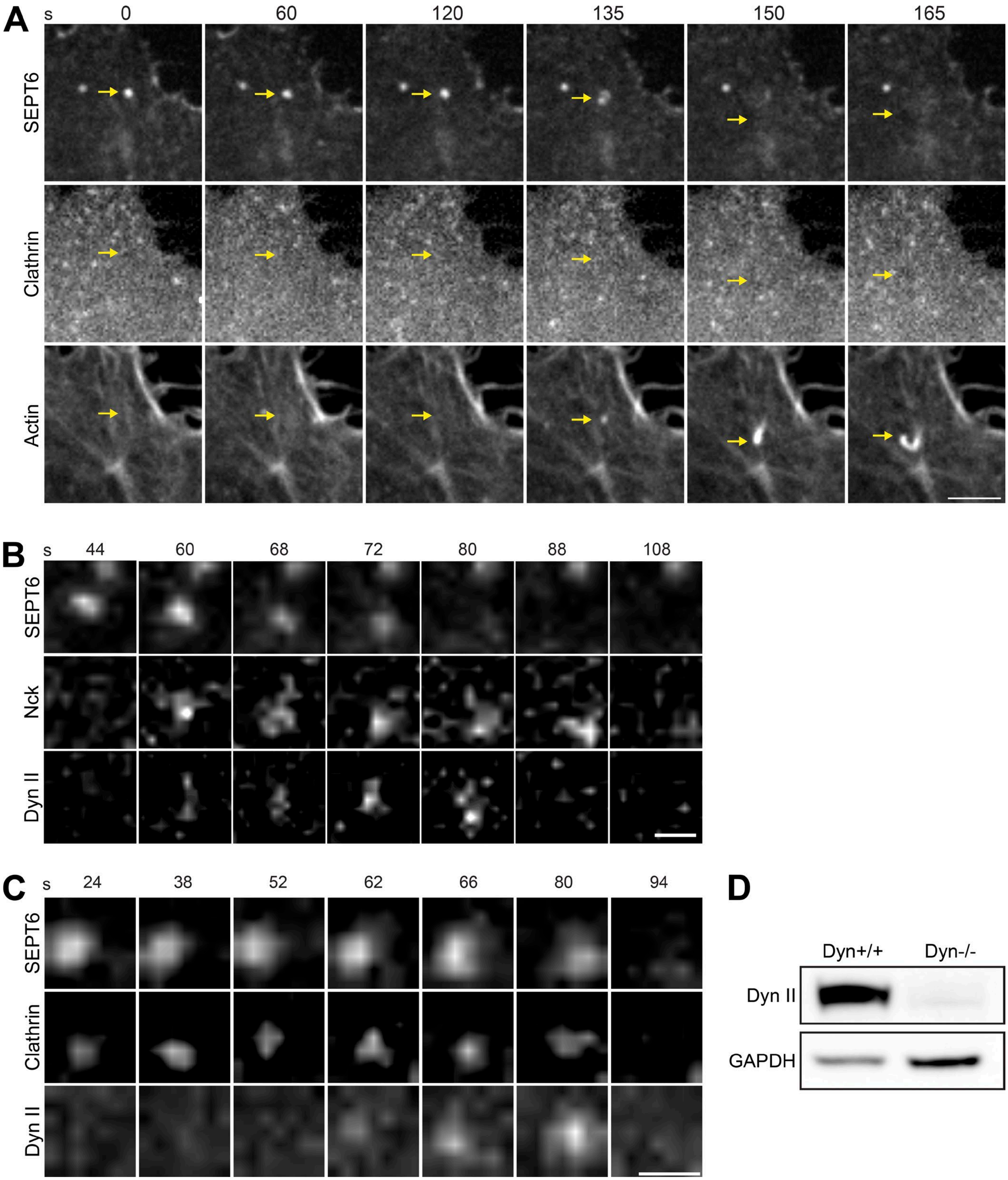

Figure S4. **Nck and dynamin are recruited when clathrin and septin are lost from the CEV. (A)** Video stills taken from live cell imaging of WR-infected HeLa cells expressing mCherry clathrin light chain (Clathrin), GFP-SEPT6, and Lifeact-iRFP (Actin). The yellow arrow highlights a virus recruiting septin but no clathrin before actin tail formation. See Video 5. Bar, 5 µm. **(B)** Video stills of the association of GFP-SEPT6, iRFP–dynamin (Dyn) II, and RFP-Nck with CEV in WR-infected HeLa cells at the indicated time. See Video 7. **(C)** Video stills of the association of GFP-SEPT6, mCherry-clathrin light chain (Clathrin), and iRFP–dyn II with CEV in WR-infected HeLa cells at the indicated time. See Video 8. **(D)** Immunoblot analysis confirming that tamoxifen treatment of Dyn$^{-/-}$ fibroblasts leads to loss of dyn II. Bars: (B and C) 1 µm.

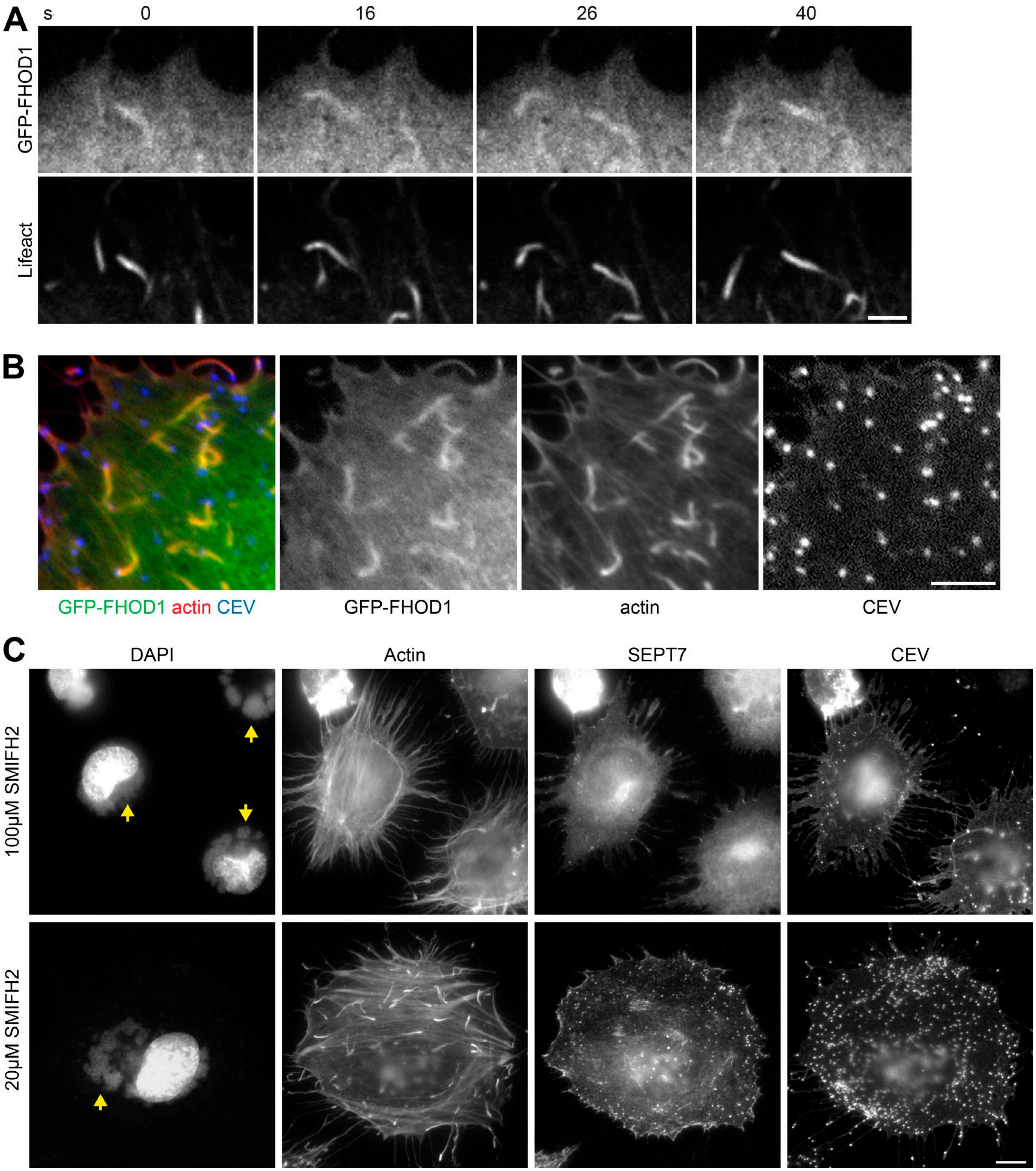

Figure S5. **Dynamin promotes loss of septin from CEV. (A)** Video stills showing the association of GFP-FHOD1 to actin tails in live WR-infected cells. Bar, 2 µm. See Video 10. **(B)** Representative fixed immunofluorescence images showing GFP-FHOD1 is recruited to actin tails induced by CEV in WR-infected cells. Bar, 5 µm. **(C)** Representative immunofluorescence images showing the effect of 100 µM and 20 µM SMIFH2 for 30 min on HeLa cells infected with WR for 8 h. The yellow arrows point to perinuclear viral factories. Bar, 10 µm.

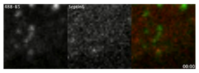

Video 1. **Septins are recruited after virus fuses with plasma membrane.** The white arrowhead highlights the recruitment of mCherry-SEPT6 to a CEV that is already labeled with extracellular Alexa Fluor 488–B5 antibody in a WR-infected HeLa cell. The time in minutes and seconds is indicated.

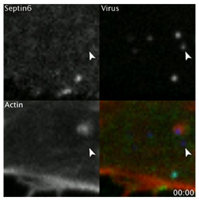

Video 2. **Vaccinia recruits septins before actin tail formation.** The white arrowhead highlights the transient recruitment of GFP-SEPT6 to WR expressing A3-RFP (Virus) in HeLa cells stably expressing Lifeact-iRFP (Actin). Septin is recruited to vaccinia in the cell periphery and subsequent actin tail formation displaces septin from the virus. The time in minutes and seconds is indicated.

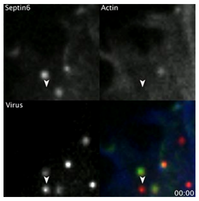

Video 3. **Septin and actin tails compete at the virus.** The video shows the transient recruitment of GFP-SEPT6 to WR virus expressing A3-RFP (Virus) in HeLa cells stably expressing Lifeact-iRFP (Actin). The white arrowhead highlights a virus where septin is initially lost but returns when actin-based motility of the virus ceases. The time in minutes and seconds is indicated.

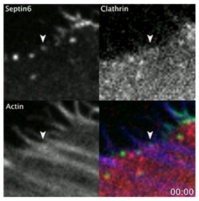

Video 4. **Septin recruitment precedes that of clathrin.** The white arrowhead highlights the transient recruitment of GFP-SEPT6, followed by mCherry-clathrin light chain (Clathrin) to CEV in HeLa cells stably expressing Lifeact-iRFP (Actin). Septin is recruited before clathrin but both are lost on actin tail initiation. The time in minutes and seconds is indicated.

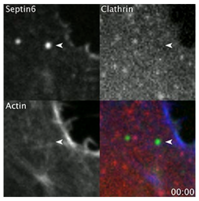

Video 5. **Clathrin does not always follow septin recruitment.** The video shows an example of the transient recruitment of GFP-SEPT6 to CEV in the absence of clathrin recruitment before actin tail formation in HeLa cells stably expressing Lifeact-iRFP (Actin) and transiently expressing mCherry-clathrin light chain (Clathrin). The time in minutes and seconds is indicated.

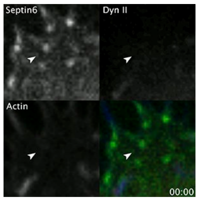

Video 6. **Septin is lost upon dynamin recruitment.** The white arrow head highlights the transient recruitment of GFP-SEPT6 to CEV in WR-infected HeLa cells stably expressing GFP-SEPT6, Lifeact-iRFP (Actin), and mCherry–dyn II. Septin is lost when dyn II arrives at the virus. Dyn II recruitment coincides with a burst of actin polymerization. The time in minutes and seconds is indicated.

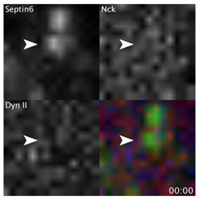

Video 7. **Nck is recruited together with dynamin.** The video shows the transient recruitment of GFP-SEPT6 to CEV in WR-infected HeLa cells stably expressing iRFP–dyn II and transiently expressing RFP-Nck1. Nck1 recruitment coincides with dyn II recruitment and the loss of septin. The time in minutes and seconds is indicated.

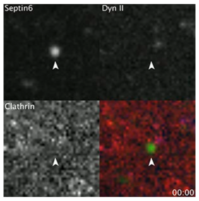

Video 8. **Clathrin is lost from virus after septin.** The video shows the transient recruitment of GFP-SEPT6 to CEV in WR-infected HeLa cells stably expressing iRFP–dyn II and transiently expressing mCherry-clathrin light chain (Clathrin). Septin and then clathrin are lost from virus when dyn II arrives. The time in minutes and seconds is indicated.

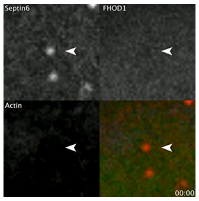

Video 9. **GFP-FHOD1 is not recruited to CEV as septin is lost.** The video shows that GFP-FHOD1 is not recruited to CEV when actin displaces mCherry-SEPT6 from WR in HeLa cells stably expressing Lifeact-iRFP (Actin). The time in minutes and seconds is indicated.

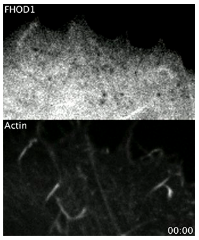

Video 10. **GFP-FHOD1 is recruited to actin tails.** The video shows that GFP-FHOD1 is weakly recruited to vaccinia-induced actin tails in HeLa cells stably expressing Lifeact-iRFP (Actin). The time in minutes and seconds is indicated.
